# Time-lapse RESOLFT nanoscopy empowered by deep learning

**DOI:** 10.1038/s41467-026-78580-z

**Published:** 2026-10-11

**Authors:** Guillaume Minet, Anirban Ray, Francesca Pennacchietti, Giovanna Coceano, Florian Jug, Ilaria Testa

**Affiliations:** 1 Department of Applied Physics and Science for Life Laboratory, KTH Royal Institute of Technology, Stockholm, Sweden; 2 Fondazione Human Technopole, Milan, Italy; 3 Technische Universität Dresden, Dresden, Germany

**Keywords:** Super-resolution microscopy, Super-resolution microscopy

## Abstract

Capturing the dynamic interplay of proteins and organelles within cells requires microscopy methods with high spatial and temporal resolution. Reversible Saturable OpticaL Fluorescent Transition (RESOLFT) demonstrated great potential for live cell nanoscopy by using patterned light and reversibly switching fluorescent probes. Despite the latest improvement in both switching probes and optical schemes, the duration and frame rate of the time-lapse are still limited by photobleaching and spatial sampling steps. Here, we present two distinct strategies based on deep learning to restore low signal-to-noise-ratio (SNR) or under-sampled images pushing the experimental boundaries of RESOLFT. We demonstrate the capability of Hierarchical DivNoising (HDN) to restore low-SNR RESOLFT data while maintaining full spatial resolution, both in supervised and unsupervised settings. This allows a 10-fold reduction of light dose per frame, and up to 5 times longer imaging. Then, we present Fast-MoNaLISA, a method that reduces the acquisition time of any point-scanning systems. This allows a 4-fold increase in frame rate, and a reduction of scanning artefacts. Overall, our methods open up possibilities for dynamic data acquisition at the sub-organelle level, such as vesicles budding in and out from intact mitochondria, or the actin meshwork formation and dissociation.

## Abstract (web summary)

The authors use two deep-learning methods for RESOLFT nanoscopy: HDN denoising enables five-fold longer live-cell imaging, while spatial upsampling with temporal context restores subsampled data for fourfold higher frame rates and reduced artifacts.

## Introduction

Fluorescence nanoscopy has revolutionized our understanding of subcellular organization by enabling the specific visualization of organelles and protein complexes with nanoscale resolution^1^. However, monitoring molecular dynamics in living cells at sub-70 nm spatial resolution remains challenging due to photobleaching and limited acquisition speed.

The majority of fluorescence nanoscopy techniques achieving sub-70 nm resolution rely on ON–OFF state transitions to generate molecular sparsity. Among the available probes, reversibly switchable fluorescent proteins (rsFPs)^2–5^ are well-suited for live-cell imaging because they can be switched using relatively low light intensities (W-kW/cm^2^) to switch between states. Reversible Saturable OpticaL Fluorescent Transition (RESOLFT)^6–8^ exploits these properties to achieve whole-cell time-lapse imaging with sub-70 nm resolution^9–11^. However, the finite photoswitching endurance of rsFPs, often referred to as photoswitching fatigue, ultimately limits the duration of observation.

In addition, the temporal resolution of RESOLFT imaging is constrained by both the time required for rsFP switching and fluorescence excitation, and by the number of scanning positions used to record an image. The latter is typically chosen according to the Nyquist–Shannon sampling criterion, such that acquisition time scales directly with the sampling density. Reducing the spatial sampling, therefore, offers a straightforward route to faster imaging, enabling the observation of rapid biological processes while minimizing motion artifacts.

Here, we apply deep-learning-based approaches to address both limitations. First, we use image denoising to reduce the illumination dose required for imaging and thereby mitigate photobleaching. Specifically, we propose the use of Hierarchical DivNoising (HDN)^12^, which outperforms existing denoising methods in preserving spatial resolution in both supervised and unsupervised settings. Second, we develop a framework for restoring sparsely sampled acquisitions, enabling increased temporal resolution through computational upsampling. We implement both single-frame and temporal training strategies and demonstrate that incorporating temporal information further improves reconstruction accuracy.

**Fig. 1:**
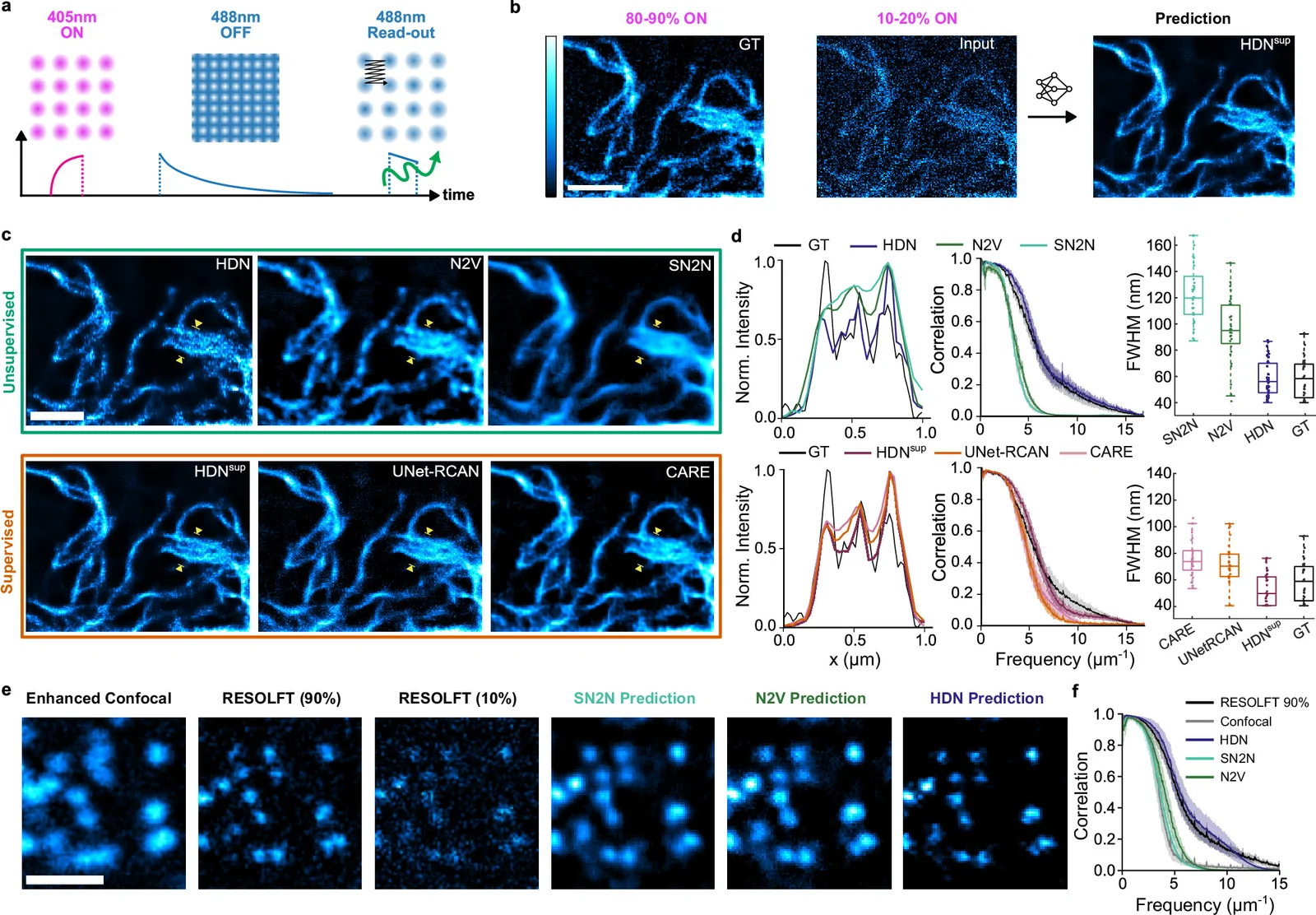
Denoising of low ON-switching MoNaLISA images. **a** Sequence of MoNaLISA imaging. Black arrows shown between 488 nm read-out foci represent the scanning pattern used during imaging. **b** MoNaLISA imaging of fixed U2OS cells endogenously expressing vimentin-rsEGFP2 for: 80-90% ON-switching (left), 10-20% ON-switching (middle), and 10–20% ON-switching denoised with our proposed method. Representative images from a test dataset of *n* = 5 cells with similar results. Scale bar, 1 µm. **c** Denoised predictions of 10–20% ON-switching MoNaLISA for HDN, N2V and SN2N as unsupervised methods (top row) and HDN^sup^, UNet-RCAN and CARE as supervised ones (bottom row). Scale bar, 1 µm. **d** Resolution analysis of denoising results for unsupervised (top row) and supervised methods (bottom row). Line profile example with normalized intensity (left column), single-image Fourier Ring Correlation (mean ± std, *N* = 3 images, middle column), and FWHM fit of line profiles of 30 filaments. Box plots show the mean (centre line), 25th and 75th percentiles (box bounds), and whiskers show data points within 1.5 times the interquartile range. **e** Live HeLa cells expressing fusion protein HIV Gag-IM-rsEGFP. From left to right: enhanced confocal (no OFF-switching), RESOLFT with 90% ON-switching, RESOLFT with 10% ON-switching and predictions of the 10% ON-switching RESOLFT image with unsupervised methods: SN2N, N2V and HDN. Scale bar, 1 µm **f** Single-image Fourier Ring Correlation (mean ± std, *N* = 8 images). Color bar indicates normalized image intensity independently scaled for display.

We focus our work on RESOLFT, which has in its favor a simpler image formation with no enforced prior on the detected signal and minimal cross-talk. This allows a robust image reconstruction, less prone to reconstruction artefacts in very noisy conditions, and offers a straightforward framework for the upsampling training. More specifically, we used the MoNaLISA implementation^9^, which represents the current state-of-the-art with parallelized RESOLFT imaging, combining multi-foci patterns for both ON-switching and read-out along with superimposed periodic line patterns for OFF-switching (Fig. 1a), achieving live-cell recordings with 45-60 nm spatial resolution at a frame rate of ~0.5 Hz in a ~ 40 × 40 µm^2^ FOV.

## Results

### Restoration of low ON-switching images with HDN

To mitigate photobleaching, we aimed at reducing the ON-switching intensity, which modulates the number of observed fluorescent molecules and therefore the intensity of the recorded signal^13^. In a typical RESOLFT experiment, 80–100% of the rsFPs are ON-switched, thus maximizing the Signal-to-Noise Ratio (SNR). Here, we propose to ON-switch only 10–20% of the rsFPs, thus acquiring low-SNR images, and to restore high-SNR images afterward with post-processing denoising. This not only minimizes photobleaching, but also mitigates potential phototoxicity that arises at the short irradiation wavelength used for ON-switching, typically 405 nm^14^.

Denoising has given rise to numerous deep-learning-based methods, including both supervised approaches that rely on paired clean and noisy data and unsupervised (or self-supervised) methods that learn to denoise without explicit clean targets. Although unsupervised methods are especially attractive for live cell nanoscopy because paired datasets are notoriously difficult to obtain, supervised methods generally offer superior performance. We thus aimed to find a denoising method that leverages supervised learning whenever high-quality ground truth is available, while still maintaining robust performance in unsupervised settings when it is not. The ideal strategy should also provide effective noise reduction without compromising the spatial resolution.

To find the best method, we systematically assessed the spatial resolution of predicted and experimentally generated GT images in two ways: by measuring the full width at half-maximum (FWHM) of fluorescently labeled vimentin filaments and calculating the image’s Fourier Ring Correlation (FRC). The images of the very same sample region were acquired with low (~30 W/cm^2^) and high (~300 W/cm^2^) ON-switching energy (Fig. 1b). This paired dataset was used to train the supervised deep learning networks and evaluate the performance of both supervised and unsupervised methods (Fig. 1c).

We found that unsupervised (or self-supervised) methods such as Noise2Void (N2V)^15^ or Self-Inspired Noise2Noise (SN2N)^16^ do not maintain the spatial resolution: the average FWHM is 120 nm for SN2N, and 93 nm for N2V, while the GT’s average FWHM is 58 nm (Fig. 1d). The same can be concluded from the FRC curves, where the curves of SN2N and N2V are clearly shifted to the lower frequencies compared to the GT’s (Fig. 1d). Supervised methods like Content-Aware Image Restoration (CARE)^17^ or the more recent UNet-RCAN^18^ perform better than the unsupervised but still tend to smooth cellular structures and lose the finest features, resulting in higher average FWHM than the GT (73 nm for CARE, 70 nm for UNet-RCAN) and FRC curves dropping at lower frequencies.

Importantly, the limited performance of the supervised methods comes from the GT’s limited quality, and not the network architecture itself. In fact, a small degradation of the GT’s quality results in a strong increase of the smoothing effect in the prediction (Supplementary Fig. S1 and Supplementary Note 1), suggesting that a perfect GT would suppress the smoothing. This highlights the difficulty of using supervised methods, since experimentally obtained GT, even at high ON-switching, will always be slightly corrupted by noise.

Therefore, we propose here to use HDN^12^, an unsupervised denoising technique which leverages a Ladder Variational Autoencoder (LVAE) architecture combined with an experimentally obtained noise model, and its supervised version HDN^sup^.

The LVAE learns a hierarchical structural prior of the biological data, capturing features from coarse shapes to fine textures. Importantly, it is designed to represent a multi-solution prior, and is trained to predict diverse solutions, allowing the model to represent uncertainty about the clean signal, instead of collapsing to one deterministic output. The level of diversity encoded in the latent space must be fine-tuned to balance between the prediction sharpness and the denoising quality (Supplementary Fig. S3 and Supplementary Note 2).

The experimental noise model is the feature enabling unsupervised training. It describes the conditional probability $p({x|s})$ of observing a clean signal value s given a noisy observation x of the imaging system. During training, for each predicted signal ${\boldsymbol{s}}$, the loss scores the likelihood of the input x under the noise process $p({x|s})$, thus constraining the reconstruction to be consistent with the data’s true noise statistics. This same framework is easily adapted for supervised training (Supplementary Note 2), allowing HDN^sup^ to benefit from the clean signal knowledge, while keeping the multi-solution representation, and the tunability of the output’s diversity.

We found that, contrary to all other methods, HDN and HDN^sup^ maintain the spatial resolution with average FWHMs of 56 nm and 54 nm, respectively. This is also confirmed by FRC curves: HDN closely matches the GT, and although HDN^sup^ is dropping slightly below the GT, it is the most shifted towards the higher frequencies compared to other supervised methods. This superiority of HDN and HDN^sup^ in terms of spatial resolution is partly due to the possibility of fine-tuning the level of diversity encoded in the latent space, which reduces the smoothing effect seen in other denoising methods. However, like other supervised methods, HDN^sup^ performance is still tightly linked to the GT’s quality (Supplementary Fig S1).

The strong performance, both in supervised and unsupervised settings, highlights the adaptability of the HDN framework to varying data constraints: HDN^sup^ offers superior restoration, especially on inputs with the lowest SNR (see Supplementary Fig. S2), but only if sufficient GT quality is available, while unsupervised HDN is a robust alternative.

To test HDN on a sparser structure, we used live HeLa cells expressing an HIV Gag-IM-rsEGFP2 fusion protein, which forms clusters of virus-like particles. For the training, we acquired MoNaLISA timelapses at ~10% ON-switching and trained directly unsupervised HDN on this data. For evaluation, we acquired a different test dataset, with images at 10% and 90% ON-switching, as well as diffraction-limited, enhanced-confocal images for comparison. We found that HDN accurately restores this dot-like structure and preserves the super-resolved details that cannot be seen in the enhanced confocal modality, contrary to other unsupervised methods (Fig. 1e) and is also confirmed by the FRC curves (Fig. 1f).

However, prediction accuracy will decrease for images at very low SNR, and this is why the ON-switching level must be chosen and validated carefully to ensure reliable predictions. Finding the right SNR will depend on many experimental parameters, such as the labeling density and the probe’s brightness. For HIV Gag-IM-rsEGFP2, we found that ~10% ON-switching is optimal, while ~5% ON-switching is too low for reliable predictions (Supplementary Fig. S5).

### Prolonged time-lapse MoNaLISA imaging

**Fig. 2:**
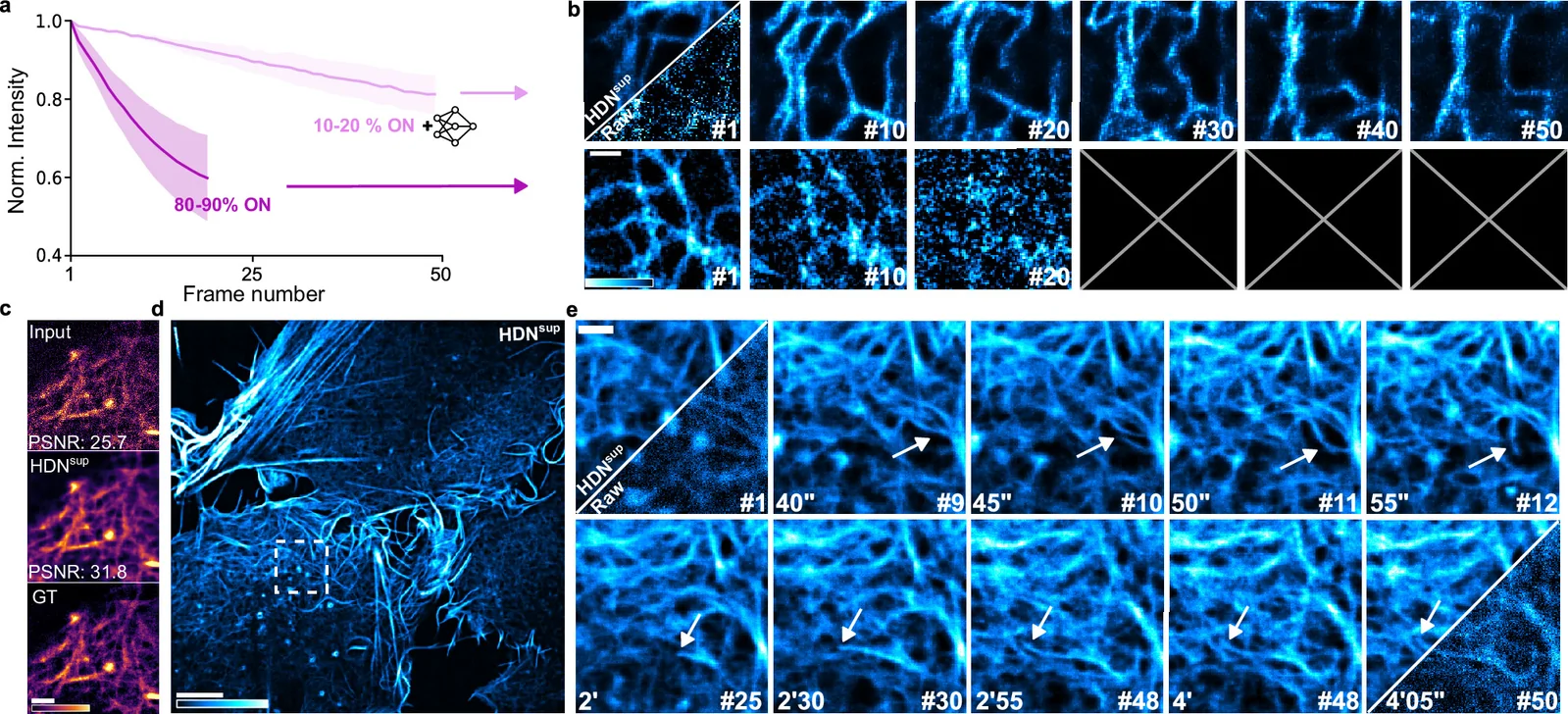
Prolonged MoNaLISA imaging. **a** Photobleaching profile for 80–90% ON-switching (dark magenta) and 10–20% ON-switching (light magenta) imaging of U2OS cells endogenously expressing vimentin-rsEGFP2 (mean ± std, *N* =  5 cells). **b** Timelapse imaging with 10–20% ON-switching (top row) and 80–90% ON-switching (bottom row). The 80–90% ON-switching time-lapse was stopped after the 20th frame because of the photobleaching. Scale bar, 500 nm. **c** Representative raw input image, HDN^sup^ prediction, and corresponding GT image of the Actin meshwork in fixed U20S cells expressing LifeAct-rsEGFP2, from a test dataset of *n* = 8 cells. Input was acquired with ~ 50% ON-switching. GT is an average of 5 frames at ~ 90% ON-switching. Scale bar, 1 µm. **d** Example of full FOV HDN^sup^ prediction, representative of *n* = 3 time lapses acquired independently with similar results. Scale bar, 5 µm. **e** Magnified region (see inset h) showing the actin meshwork evolving over 50 frames, acquired every 5 s. White arrows highlight meshwork movements. For the first and last frame, the lower right corner shows the raw image data. Scale bar, 1 µm. Color bar indicates normalized image intensity independently scaled for display.

We next investigated whether our approach enables prolonged time-lapse imaging in living cells. Given its superior performance under low signal-to-noise conditions, we applied the supervised HDN^sup^ network, trained on the fixed-cell vimentin dataset, to live-cell recordings. Since the images were recorded with ~10 times less energy of UV-Vis light, photobleaching was substantially reduced, resulting in up to ~5 times longer time-lapse imaging compared to conventional RESOLFT done with higher ON-switching light (Fig. 2a, b). Of note, these experiments were performed with endogenous levels of expression of vimentin molecules. For brighter structures, such as mitochondria labeled with rsEGFP2 targeting the outer membrane, we used the unsupervised HDN to extend timelapses over more than 100 frames (Supplementary Fig. S6 and Supplementary movie 1).

Finally, we used HDN^sup^ to image complex and interlaced structures such as the actin meshwork, which is often too dim to be imaged for more than a few frames in conventional RESOLFT. We first acquired a high-quality training dataset on fixed cells expressing LifeAct-rsEGFP2. After validating the capability of HDN^sup^ to accurately restore the meshwork (Fig. 2c), we applied it to live cells and were able to observe the dynamics of the meshwork for at least 50 frames (Fig. 2d, e and Supplementary movie 2).

### Fast-MoNaLISA–single-frame training

**Fig. 3:**
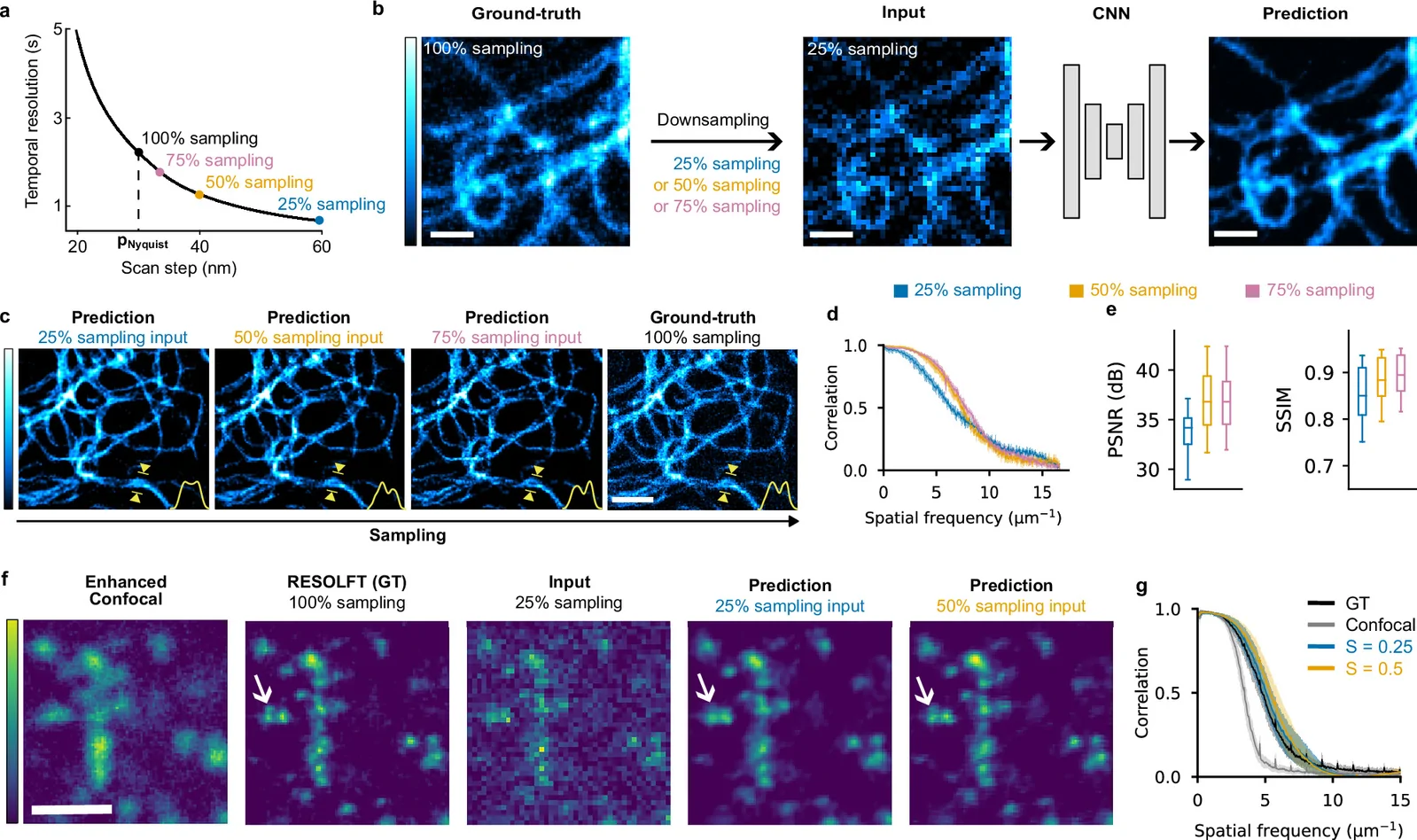
Fast-MoNaLISA–single frame training and results. **a** Temporal resolution dependency over the scanning pixel size. Frame rate calculated for a multi-foci period of 625 nm and a dwell time of 5 ms. **b** Training setup for Fast-MoNaLISA. Starting from a Nyquist-sampled image (the GT), we select a subset of pixels to create a subsampled input of a given sampling ratio ***S*** (75%, 50% or 25%). Images are representative examples from the test dataset (*n* = 3 cells). Scale bar, 1 µm. **c** Fast-MoNaLISA results examples on U2OS cells endogenously expressing vimentin-rsEGFP2, across sampling ratios. The line profile across the orange arrows is reported in orange. Scale bar, 1 µm. **d** Relative FRC between predictions and GT on the vimentin dataset (mean ± std, *n* = 3 cells). **e** PSNR and SSIM performances for all sampling ratios on the vimentin dataset (*n* = 3 cells). Centre line: mean, box bounds: 25th and 75th percentile, whiskers: extreme data points within 1.5 time the interquartile range. **f** Diffraction-limited confocal image, and fast-MoNaLISA results examples on live HeLa cells expressing fusion protein HIV Gag-IM-rsEGFP. White arrows highlight an improvement in the 50% sampling prediction over the 25% sampling prediction. Similar results are found on the whole test dataset (*n* = 17 cells). Scale bar, 1 µm **g** Single-image Fourier Ring Correlation on the Gag-IM particles dataset (mean ± std, *n* = 17 images). Color bar indicates normalized image intensity independently scaled for display.

To enable the visualization of faster live-cell dynamics, we explored strategies to accelerate MoNaLISA image acquisition. Since acquisition time is determined by the number of scanning steps, we reasoned that sparse sampling combined with image restoration could substantially increase imaging speed while preserving image quality (Fig. 3a). The optimal step size *p* is typically chosen according to the Nyquist-Shannon theorem to avoid aliasing or redundant information, and it is twice the frequency limit of the system, which is roughly half of the expected resolution, thus *p* ~ 20–30 nm in RESOLFT imaging.

Current computational methods aiming at improving temporal resolution propose to predict new timepoints between existing recording^19^, or to recover dynamics happening during a multi-shot imaging^20^. However, they do not aim at reducing the recording time. On the hardware side, smart acquisition schemes have been successfully applied to point-scanning STED^21^ or RESOLFT^22^, but they rely on the fast response of single-point detectors and are hardly applicable to parallelized, camera-based systems.

Here, we introduce a new method, Fast-MoNaLISA, to reduce the acquisition time per frame by skipping scanning steps and then restoring subsampled images with an upsampling network. This has the significant benefit of improving temporal resolution at the same illumination cost. Although a similar approach was applied to diffraction-limited confocal microscopy^23^, it was never shown to work for sub-100 nm nanoscopy images. One specificity of our method is that we reduce the sampling while keeping the same OFF-switching dose, therefore still imprinting the same resolution in the sample.

We first aimed at studying the up-sampling restoration in static settings, i.e., providing a single time point to the network, which we called single-frame training.

Since each pixel of a reconstructed MoNaLISA image can be mapped to one specific scanning position, the training of the upsampling network is straightforward (Fig. 3b and Supplementary Fig. S9). Starting from a Nyquist-sampled image, our GT, we apply a down-sampling step by removing a subset of pixels, thus creating a subsampled image, which is the input into the network. This procedure is fully equivalent to acquiring experimentally subsampled MoNaLISA data and reconstructing the image, meaning that the training inputs accurately match the subsampled images used during inference of real fast acquisitions.

Importantly, the down-sampling does not include any blurring, as is usual in a common upsampling network training setup. However, because the retained input pixels have the exact same values as the corresponding GT pixels, including any residual noise present in the GT, we randomized the pixel-selection step during training. This prevents the network from learning a fixed spatial pattern of retained pixels, and avoids the patterned noise observed with deterministic subsampling (Supplementary Fig. S10) and allows a built-in denoising effect instead.

By tuning the proportion of removed pixels, we can choose the sampling ratio, which we denote *S*. As *S* gets smaller, the frame-rate gain increases, but the restoration task gets harder, as less information is provided to the network. We studied three sampling ratios of *S* = 75%, *S* = 50%, and *S* = 25%, corresponding to increases of 1.3-fold, 2-fold, and 4-fold in frame rate, respectively.

For the network architecture, we chose the previously mentioned UNet-RCAN architecture, but kept the last upsampling layer of the original RCAN^24^. We chose this network because RCAN was initially designed for upsampling tasks, and we found that keeping the first U-Net network helps the network to converge.

We first tested our approach on the same structure used previously, fixed cells expressing vimentin-rsEGFP2, with an ideal SNR, corresponding to >80% ON-switching level. Importantly, UNet-RCAN does not create any smoothing effect, and maintains the spatial resolution in terms of FWHM and FRC curves, for all sampling levels (Supplementary Fig S12). This is because, contrary to the supervised denoising task, the upsampling training setup is not limited by the GT’s quality: the subsampled input is created directly from the fully sampled GT and has a perfect matching target. Indeed, we found that if the target is replaced by another fully sampled acquisition, with independent noise, the up-sampled prediction is oversmoothed (Supplementary Note 1 and Supplementary Fig. S11).

We then evaluated the prediction accuracy by comparing the predictions with the fully sampled GT. As expected, we found that the prediction improves when the sampling ratio ${\boldsymbol{S}}$ increases, especially from *S* = 25% to *S* = 50%. This can be seen from the example line profile (Fig. 3c, insets), the relative FRC between the prediction and the GT (Fig. 3d), as well as peak signal-to-noise ratio (PSNR) and structural similarity index measure (SSIM) (Fig. 3e). However, we found that higher sampling ratios are not always beneficial in the case of lower SNR (Supplementary Fig. S13). We hypothesize that this is due to a lower denoising effect for higher sampling ratios, which becomes more important when the SNR decreases. Importantly, the gain from *S *= 50% to *S *= 75% resulted in minor or no change for all SNR levels, suggesting that sampling half of the information is enough to restore subsampled images with the highest accuracy. For lower sampling (*S* = 25%), the network still allows for the recovery of most of the information, but there is more risk of prediction errors, especially for low SNR inputs.

We found similar results on the HIV Gag-IM virus-like particles (Fig. 1e). While the FRC shows that the resolution is maintained for both *S* = 25% to *S* = 50% (Fig. 3f), some of the finest details are more accurately reconstructed with *S* = 50% (Fig. 3e, white arrows).

The first part of our study on single-frame training demonstrates two points. First, Fast-MoNaLISA performs best on high-SNR images and should be used at regular ON-switching levels, i.e., ~80–100%, which remains compatible with our general aim of light-dose reduction, since the sub-sampling intrinsically reduces it. Second, the choice of sampling ratio reflects a trade-off between imaging speed and prediction reliability. At low sampling ratios, especially *S* = 25%, the reduced spatial information makes fine or isolated structures more ambiguous to reconstruct. The learned prior can then bias the prediction toward the most likely structure in the training distribution, leading to suppression or inaccurate reconstruction of faint details, rather than hallucination of non-existing structures. For single-frame prediction, *S* = 50%, therefore, it appears to be the most robust compromise between speed and fidelity.

### Fast-MoNaLISA–multi-frames training

**Fig. 4:**
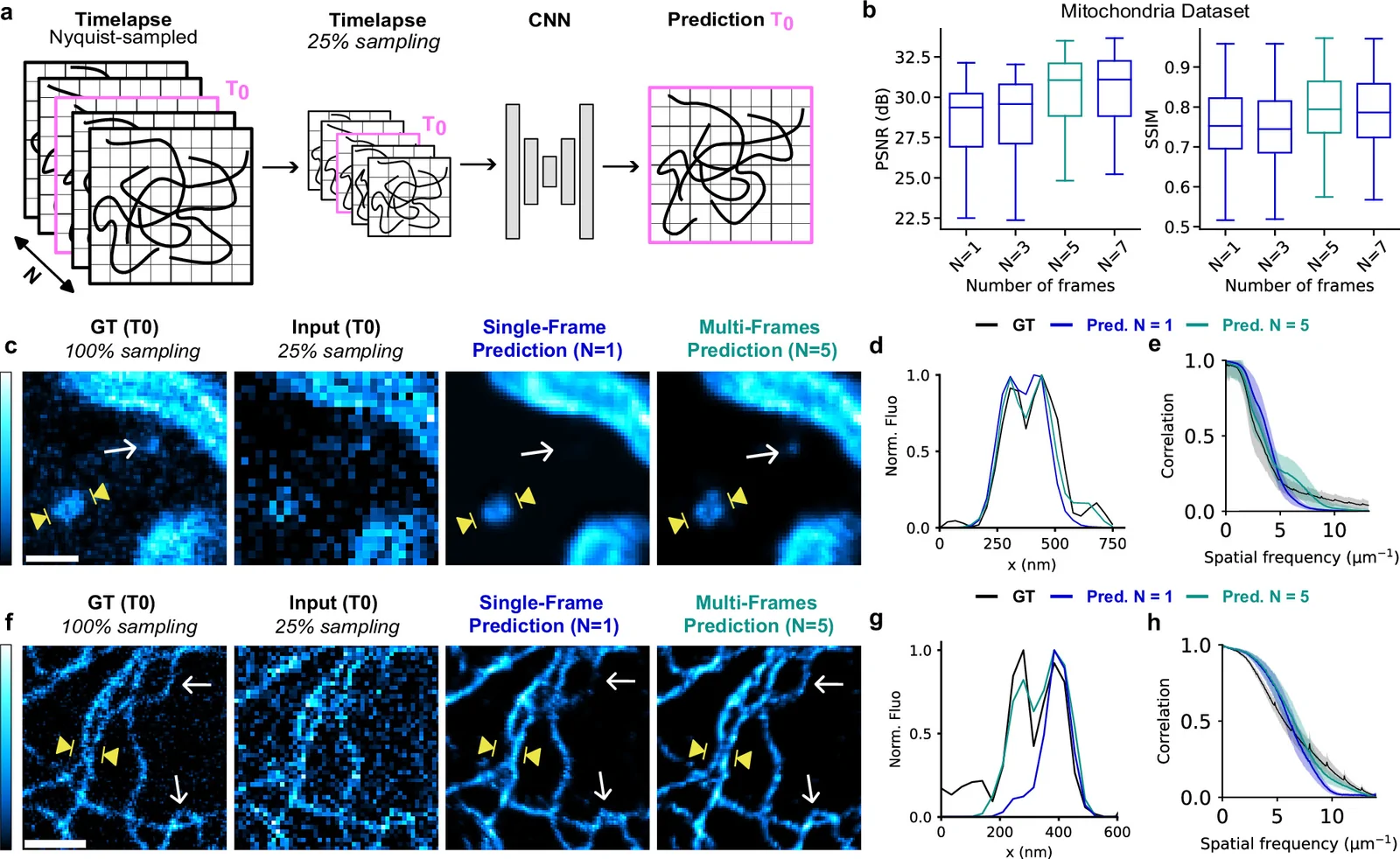
Multi-frames Fast-MoNaLISA training and results. **a** Multi-frames training concept: a subsampled (***S*** = 25%) time-lapse with 5 consecutive frames is used to restore the centre timepoint **T**_**0**_. The surrounding timepoints give temporal context and help the restoration task. **b** PSNR and SSIM results for the mitochondria dataset, obtained on U2OS cells expressing rsEGFP2-Omp25. Metrics are calculated between the GT and predictions for different numbers of frames of the input timelapse (*n* = 100 images). Centre line: mean, box bounds: 25th and 75th percentile, whiskers: extreme data points within 1.5 times the interquartile range. **c** Example of prediction improvement between single and multi-frame predictions on the mitochondria dataset. The white arrow highlights a faint vesicle that is lost in the single-frame prediction but correctly restored in the multi-frames. Scale bar, 1 µm. **d** Line profile with normalized intensity along orange arrows in **c**. **e** Single- Image Fourier Ring Correlation comparing the performances of single and multi-frames training on the mitochondria dataset (mean ± std, *n* = 100images.) **f** Examples of prediction on live U2OS Cells endogenously expressing vimentin-rsEGFP2 with single and multi-frames predictions on the mitochondria dataset. Scale bar, 1 µm. **g** Line profile with normalized intensity along orange arrows in (**f**). **h** Single-Image Fourier Ring Correlation comparing the performances of single and multi-frames training on the vimentin dataset (mean ± std, *n* = 80 images). The color bar indicates the normalized image intensity independently scaled for display.

Since the goal of Fast-MoNaLISA is to image the rapid dynamics of trafficking molecules and organelles within cells, we hypothesized that imaging data containing the temporal evolution of the imaged cellular structure, i.e., a temporal context, could improve the accuracy of the predictions, especially for the lower sampling *S* = 25%. To test this idea, we implemented a variation of our previous training, which we call multi-frames training, where we use a sub-sampled timelapse as an input instead of a single snapshot. For each timepoint, the subsampled image is generated by applying the same randomized downsampling step previously used in the single-frame training. The network is then trained to predict one single timepoint, the middle timepoint *T*_*0*_ of the time-lapse, but now learning additional information provided by the new temporal context of the input (Fig. 4a).

We tested the multi-frame training on vimentin filaments, mitochondria and Gag particles. To find the best timelapse length, we systematically trained the network for *N* = 3, *N* = 5 and *N* = 7 input frames, and evaluated both PSNR and SSIM metrics (Fig. 4b and Supplementary Fig. S14). We found that *N* = 5 frames was best for the vimentin and mitochondria datasets, while *N* = 7 frames was best for the Gag-IM dataset.

Thanks to the temporal context, the prediction’s spatial resolution and accuracy improved for all datasets, with varying degrees. More specifically, on the mitochondria dataset, it allowed for better resolution of mitochondrial-derived vesicles (MDVs) and mitochondria-vesicle contacts (Fig. 4c, d and Supplementary Fig S14), and this was confirmed by FRC curves (Fig. 4e). Additionally, the denoising effect was more accurate: small, dim vesicles are sometimes completely removed with the single-frame prediction, considered as noise, which was not the case for the multi-frames (Fig. 4c, white arrow, Supplementary Fig. S14). On vimentin filaments, in addition to improving spatial resolution (Fig. 4f–h), the temporal context helped with filament continuity (Fig. 4f, white arrows, Supplementary Fig. S14). Smaller but similar improvements were observed on the Gag-IM (Supplementary Fig. S14). As expected, the best time-lapse length and the amount of improvement gained from the multi-frame training will depend on the biological structure and its dynamics.

Therefore, we concluded that the multi-frame training pushes the limits of Fast-MoNaLISA by accurately reconstructing images sub-sampled at *S* = 25%.

### Fast-MoNaLISA enables monitoring of rapid mitochondrial dynamics

**Fig. 5:**
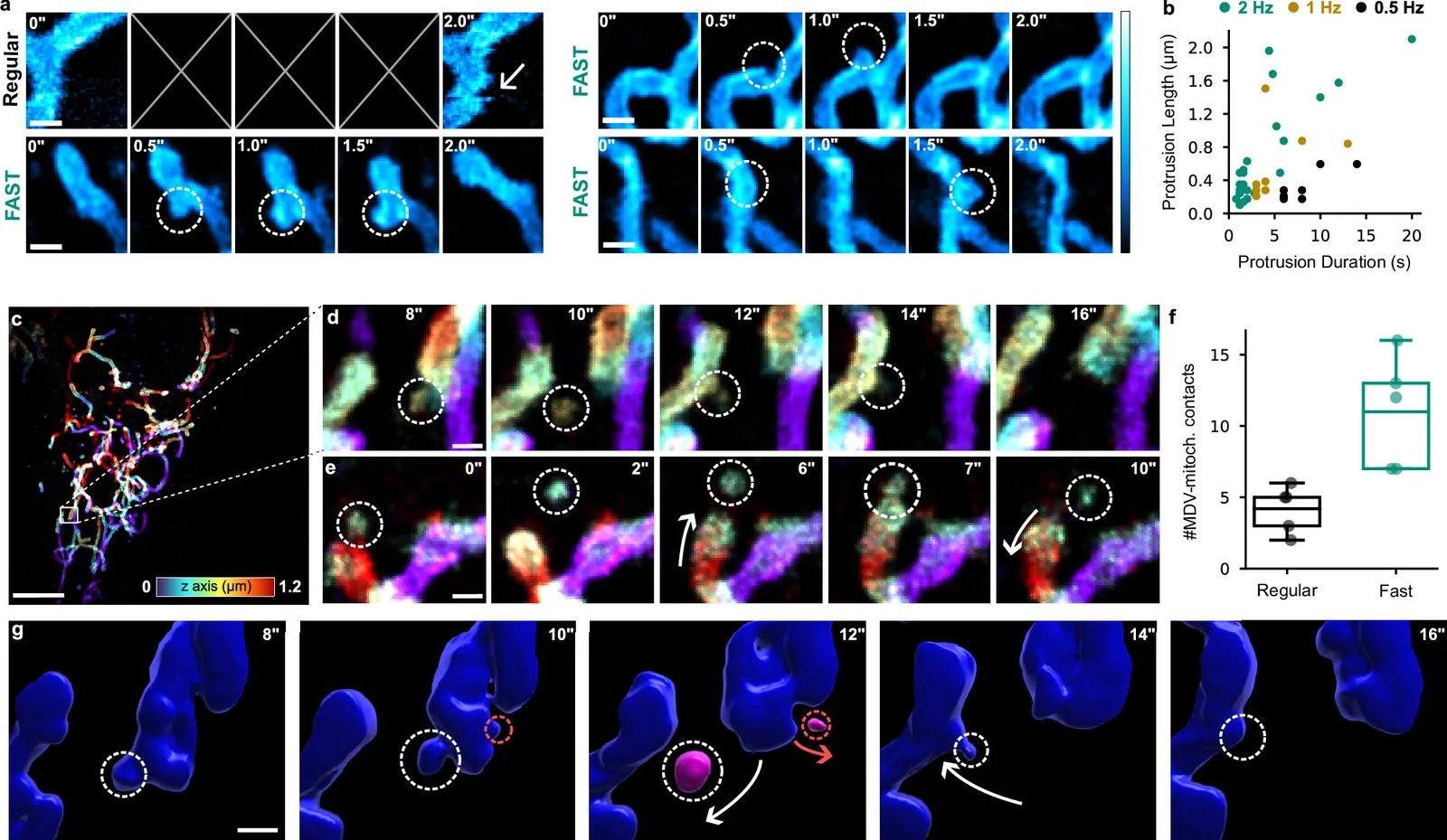
Fast-MoNaLISA application to mitochondrial dynamics. **a** Regular vs Fast-MoNaLISA imaging examples of mitochondria in U2OS cells expressing rsEGFP2-Omp25. Regular MoNaLISA images are raw images acquired at 0.5 Hz (empty gray rectangles with the cross correspond to the timepoints not accessible at this imaging speed). Fast images are multi-frame predictions of ***S*** = 25% sampled images acquired at 2 Hz. White dotted circles highlight fast protrusions happening during the time acquisition of the slow modality. Scale bar, 500 nm. Color bar indicates normalized image intensity independently scaled for display. **b** Characterization of the protrusion’s lengths and duration for 3 imaging speeds: 2 Hz (red), 1 Hz (orange), 0.5 Hz (black). **c** Full FOV of fast volumetric recordings of mitochondria, Z axis color-coded. Scale bar, 10 µm. **d** Zoomed-in time-lapse region of **c** showing an MDV budding out and contacting another mitochondrion. **e** Zoomed-in region from a different cell showing MDV re-contacting the same mitochondrion after several seconds. Scale bar, 500 nm. **f** Number of mitochondria-MDVs interactions seen per cell with Regular and Fast-MoNaLISA, *N* = 5 cells. Centre line: mean, box bounds: 25th and 75th percentile, whiskers: extreme data points within 1.5 time the interquartile range. Individual data points are also shown. **g** Volumetric modelling of region shown in **d**. White circles and arrows highlight the path of one MDV, while red circles and arrow follow a second vesicle. Top-down view, scale bar, 500 nm.

To demonstrate the utility of our approach, we used the Fast-MoNaLISA with ***S*** = 25% and a multi-frames prediction (*N* = 5 frames), to observe fast mitochondria dynamics. First, with 2D single plane imaging, we could image a full MoNaLISA FOV of ~ 35 × 35 µm^2^ at a frame rate of 2 Hz, 4 times faster than regular MoNaLISA. With mitochondria moving fast, reducing the acquisition time is key to avoiding motion artifacts (Fig.5a and Supplementary movie 3). This allowed us to observe the temporary formation of small mitochondrial membrane protrusions elongating and retracting very rapidly, clearly happening too fast to be seen at lower frame rates (Fig. 5a and Supplementary Fig. S15; Supplementary movie 4). We characterized the length and dynamics of all elongating and retracting mitochondria movements at three different frame rates: 2 Hz (fast-MoNaLISA), 1 Hz (regular MoNaLISA with smaller FOV for faster dwell time), and 0.5 Hz (regular MoNaLISA, full FOV). The 2 Hz imaging clearly allows us to detect, faster (1.5-4 s) and smaller (80-200 nm) protrusions that are completely missed in the other imaging speeds (Fig. 5c).

Mitochondria are spatially distributed throughout the whole 3D cell volume, and acquisition of images at different axial positions, as in 3D stacks, is thus often needed to comprehensively monitor and resolve the whole network of organelles. Using the Fast-MoNaLISA, we could speed up the whole 3D mitochondrial network imaging compared to conventional recordings, thus enabling volumetric acquisition rates of up to 0.5 Hz, or even 1 Hz with a smaller FOV (Fig. 5e). At this volume rate, we were able to observe a large variety of mitochondria dynamics, including the formation of MDVs budding out and to follow their fate over time. MDVs have been shown to deliver specific cargoes to different subcellular locations, such as the endo-lysosomal system^25^. More recently, it has been shown that MDVs carry functional ATP synthase and, therefore, were proposed as a potential way to deliver functional protein complexes to malfunctioning mitochondria^26^. The ability of MDVs to fuse with existing mitochondria has not been demonstrated yet and is still under debate. Fast-MoNaLISA can help investigate the fate of MDVs and the proposed ability to exchange material within the mitochondrial network. We often detect vesicles contacting multiple mitochondria prior to fusion (Fig. 5d, g and Supplementary movie 5), or a vesicle fusing in and budding out a few seconds later (Fig. 5e). Many other examples are shown in Supplementary Fig. S16.

Vesicle dynamics require volumetric imaging to properly follow a vesicle through the field of view and to differentiate between a vesicle and a 2D section of a mitochondrion’s tip. However, without the Fast-MoNaLISA, the volume rate is too slow to follow vesicle movements. We estimated that Fast-MoNaLISA enables the detection of at least 3 times more mitochondria-MDVs interactions, compared to regular MoNaLISA (Fig. 5f, Methods).

## Discussion

In summary, we presented a hybrid computational-experimental approach capable of providing a comprehensive time-lapse of whole cells at high spatial resolution (x-y ~ 60 nm), with a frame rate of ~2 Hz in 2D, or a volume rate of 0.5-1 Hz in 3D, adding new imaging ability to a diverse set of structures from the cytoskeleton to mitochondrial vesicles. Longer-duration and higher-frame-rate time-lapse imaging broadened the range of structures accessible to parallelized RESOLFT live cell nanoscopy, including dim structures which could previously only be visualised as snapshots, or fast-moving molecules that need to be monitored in 3D for longer time.

In the field of live-cell nanoscopy, many other techniques have recently benefited from computational advances that improve the balance between spatial resolution, imaging speed and light dose.

Structured Illumination Microscopy (SIM)^27^ remains one of the most established methods for fast and gentle live-cell super-resolution, and recent deep-learning-assisted methods report prolonged SIM recordings with more than 5000 timepoints^28^. However, SIM resolution gain is limited to two-fold, and does not reach sub-70 nm resolution.

Super-resolution optical fluctuation imaging (SOFI)^29^ and related fluctuation-based methods are also attractive for low-light live-cell imaging, but reaching sub-100 nm resolution requires higher-order fluctuation statistics, which typically rely on several thousand raw frames^30^. Diverse computational strategies^31,32^ have substantially reduced this frame requirement, achieving an approximately two-fold resolution improvement from as few as 8-20 frames, but these approaches have currently been applied to second-order fluctuations only.

Stimulated Emission Depletion (STED)^33^ reaches spatial resolution comparable to RESOLFT, and a recent deep-learning approach has shown that low-SNR STED images^18^ can be restored after substantially reducing pixel dwell time, enabling faster and gentler recordings. However, the STED beam averaged power (10-100 mW) is still an order of magnitude higher than the RESOLFT one (1-10 µW). Also, STED remains mostly implemented as a point-scanning microscope whose acquisition time scales linearly with the number of pixels in the field-of-view, slowing down the acquisition of large sample regions, especially when recording 3D stacks of images.

Therefore, our methods strengthen the position of parallelized RESOLFT in live-cell nanoscopy as a particularly well-suited approach for whole-cell imaging at sub-70 nm resolution, offering a balance between spatial resolution, imaging speed, field of view and light dose that remains difficult to achieve with other nanoscopy techniques.

We also believe the methods presented are applicable and can be beneficial for other imaging modalities, and we thus released LISAI^34^, a dedicated software framework, as well as training examples to facilitate application on new datasets^35^. HDN can be used to denoise any microscopy dataset and shows especially good performance in restoring nanoscopy images. It also has the advantage of being usable both in a supervised and unsupervised manner in the same overall training structure.

Similarly, our subsampled image restoration method is directly applicable to scanning-based modalities in which each reconstructed pixel corresponds to a defined scan position. In this setting, training is straightforward because subsampled inputs can be generated directly from regular Nyquist-sampled images by computationally removing pixels, avoiding the need to acquire paired datasets of subsampled and fully sampled images. This method could therefore be beneficial for increasing the acquisition rate of other scanning-based imaging techniques, such as STED or spinning-disk confocal microscopy, and is particularly applicable for camera-based detection, where the dwell time is often limited by camera readout.

Like any other deep-learning-based image restoration approach, failure cases must be considered, and the acquisition parameters should be chosen according to the desired balance between imaging duration, temporal resolution and restoration fidelity. For prolonged imaging, reducing the ON-switching level decreases photobleaching and phototoxicity, but the ultimate limit becomes the SNR required for reliable prediction. In our conditions, 10–20% ON-switching provided a good compromise for rsEGFP2-labeled structures, whereas lower values such as ~5% led to inaccurate predictions or loss of faint structures. For Fast-MoNaLISA, the sampling ratio should be chosen according to the SNR and dynamics of the sample. A sampling ratio of *S* = 50% appears to be the most robust single-frame regime, but in dynamic acquisitions, temporal context mitigates this limitation and makes *S* = 25% reliable. Overall, validation on representative datasets remains essential to find the optimal ON-switching level and sampling ratio that does not compromise prediction accuracy.

In the current implementation of HDN, the level of diversity encoded in the latent space is manually adjusted for each new dataset, making the choice dependent on empirical assessment. Future work could treat this parameter as an explicit model hyperparameter and automate its optimization against quantitative criteria. Further deploying the multi-solution nature of HDN could allow uncertainty estimates, thereby informing users about the reliability of individual predictions. Finally, in this work, we treated denoising and upsampling as separate tasks and selected the network architecture best suited to each one, with the goal of optimizing their performance independently. However, a future unified framework that jointly addresses noise reduction and subsampling restoration could be highly valuable, potentially enabling simultaneous reductions in light dose and acquisition time within a single computational pipeline.

## Methods

### Network architectures

Most of the networks were implemented within a common PyTorch framework (see code availability below) to facilitate comparison across models and tasks, including HDN, CARE, RCAN and UNet-RCA. For SN2N and N2V, we instead used their original implementations (respectively https://github.com/WeisongZhao/SN2N and https://github.com/juglab/n2v).

HDN was implemented following the architecture from the original publication (https://github.com/juglab/HDN), which uses a Ladder Variational Auto Encoder (LVAE). CARE was implemented following https://github.com/CSBDeep/CSBDeep but adapted for PyTorch. For the UNet-RCAN denoising, we started from the original PyTorch RCAN implementation (https://github.com/yulunzhang/RCAN) and modified it according to the UNet-RCAN code (https://github.com/vebrahimi1990/UNet_RCAN_Denoising). For the upsampling task, we used the same U-Net and UNet-RCAN network architectures, but with an additional upsampling block at the end. Further details can be found in Supplementary Note 3 and Supplementary Table 2.

### Network training

For CARE training, we used the MSE loss, as used in the original implementation. For all other supervised training, we used the loss function used in the original UNet-RCAN denoising, the weighted summation of Charbonnier loss and Edge loss $({L=L}_{{char}}+\alpha {L}_{{edge}})$, with α=0.05. This loss function has been shown to reduce the smoothing effect associated with MSE loss^18^. Other training parameters, such as learning rate, batch size, and number of epochs, are reported in Supplementary Table 1-2, and training curve examples can be found in Supplementary Fig. S18.

Details for HDN training and inference can be found in Supplementary Note 2.

### Dataset acquisition

We acquired several RESOLFT datasets with different roles. Paired datasets were used when a high-SNR reference image was required, either to generate the HDN noise model, to train supervised methods, or for benchmarking predictions. Paired datasets were acquired in fixed cells (see below) to avoid structural changes between corresponding images. Unpaired datasets were used for unsupervised denoising and upsampling training, where no matched high-SNR ground truth was required. In some cases, additional independent datasets were acquired only for testing.

Full description of the different datasets acquisition and training data preparation is provided in Supplementary Note 4. Dataset-specific information, including the number of images and timepoints, training, validation, and test splits, is reported in Supplementary Table 3.

### Quantitative evaluation of the prediction

For resolution assessment, we traced *N* = 30 line profiles on vimentin filaments on FIJI, with a thickness of 3 pixels. We fitted them with a Lorentzian function with a custom MATLAB script. For the FRC comparative measurements, we used the single image FRC (1FRC) method, which we validated to give similar results to two images FRC (2FRC) on our data.

PSNR and SSIM were computed using the metrics module of the Python *scikit-image* library. Both metrics assess the agreement between a predicted image and a reference image. In experimental data, the reference image inevitably contains residual noise, which reduces the maximum achievable PSNR and SSIM even for an ideal prediction of the underlying signal. Therefore, the reported values should be interpreted as conservative estimates, or lower bounds, of prediction quality. Importantly, relative comparisons remain valid when different predictions are evaluated against the same reference image (Supplementary Fig.S17).

### MoNaLISA imaging

All images were acquired on one of the two custom-built MoNaLISA setups we have. Both setups follow the original implementation, with a few minor differences. The first setup has an OFF-period pattern ***P***_***OFF***_ = 238 nm and a multi-foci pattern ***P***_***MF***_ = 714 nm. The second setup has an OFF-period pattern ***P***_***OFF***_ = 312 nm and a multi-foci pattern period ***P***_***MF***_ = 625 nm. Images were acquired on both setups interchangeably, without a systematic preference for one over the other. We used ImSwitch^36^ (https://github.com/ImSwitch) for hardware control.

Enhanced confocal images were obtained by keeping only the ON-switching and Read-out of the RESOLFT pulse and suppressing the OFF-switching pulse. We refer to this modality as “enhanced confocal” because the combined 405 nm and 488 nm excitation/readout point-spread functions provide a slight resolution improvement compared with regular confocal imaging.

### SNR quantification

The SNR quantification was done from line profile measurements, by taking the ratio of the peak value of the Gaussian over the standard deviation of the background (Supplementary Figs. S7; S8).

### Image analysis of mitochondria dynamics

For the protrusion length and duration analysis (Fig. 5a, b), we manually measured in FIJI the maximum protrusion length and counted over how many frames it lasted, for all back-and-forth movements we could observe and detect with our eyes. This was done on 5 cells for each considered speed. For the MDV-mitochondria interactions number of events (Fig. 5c–g), we counted each time we could track a vesicle and determine its fate with relative certainty. The number of events for “Regular” imaging was estimated by keeping only 1 volume for every 4 of the “Fast” imaging. This is only an estimation, because it did not account for the additional scanning artefacts of the slower scanning, which would have made the interpretation of the mitochondria-MDV contacts even harder. Therefore, we were probably overestimating the number of events for the regular imaging.

### Cell culture

Wild-type HeLa (ATCC CCL-2) and U2OS (ATCC HTB-96) cells and U2OS with endogenously tagged vimentin-rsEGFP2 (ref. Monalisa) were cultured in DMEM (Thermo Fisher Scientific; 41966029) supplemented with 10% (vol/vol) FBS (Thermo Fisher Scientific; 10270106) and 1% penicillin–streptomycin (Sigma Aldrich; P4333) and maintained at 37 °C and 5% CO2 in a humidified incubator. For transfection with LifeAct-rsEGFP2 and rsEGFP2-Omp25 plasmids (Ref. Monalisa), 2 ×105 cells per well were seeded on coverslips in a six-well plate. After 1 day, cells were transfected using Lipofectamine LTX Reagent with PLUS reagent (Thermo Fisher Scientific; 15338100) according to the manufacturer’s instructions. 24–36 h after transfection, cells were washed in PBS solution, placed with phenol-red-free DMEM or Leibovitz’s L-15 medium (Thermo Fisher Scientific; 21083027) in a chamber, and imaged.

For fixed-cell imaging experiments, cells were washed once with PBS and then fixed in 4% paraformaldehyde (PFA) in phosphate-buffered saline (PBS) (pH7.4) at room temperature for 20 min.

### Computational resources and training time

All network training was performed on a workstation equipped with an NVIDIA RTX A4000 GPU. The training time for each model is reported in Supplementary Tables 1–2. These values correspond to the wall-clock time of the training runs used in this study and should be interpreted as practical reference times rather than optimized benchmarks. In particular, batch size and GPU memory usage were not systematically optimized for every experiment, especially during early training runs, and shorter training times could likely be obtained with further hardware-specific optimization.

### Reporting summary

Further information on research design is available in the Nature Portfolio Reporting Summary linked to this article.

## Supplementary information

Supplementary Information

Description of Additional Supplementary Files

Supplementary Movie 1

Supplementary Movie 2

Supplementary Movie 3

Supplementary Movie 4

Supplementary Movie 5

Reporting Summary

Transparent Peer Review file

## Data Availability

Training datasets, trained models and the data presented are available in the following Zenodo repository: https://doi.org/10.5281/zenodo.22877255^35^.

## Code availability

The code is available at the GitHub repository: https://github.com/GuillaumeMinet/LISAI-resolft, and is archived on Zenodo at: https://doi.org/10.5281/zenodo.22893279^34^.

## References

[1] Sahl, S. J., Hell, S. W. & Jakobs, S. Fluorescence nanoscopy in cell biology. *Nat. Rev. Mol. Cell Biol.* **18**, 685–701 (2017). 10.1038/nrm.2017.71 28875992

[2] Grotjohann, T. et al. rsEGFP2 enables fast RESOLFT nanoscopy of living cells. *Elife* **1**, e00248 (2012). 10.7554/eLife.00248 PMC3534202 23330067

[3] Brakemann, T. et al. A reversibly photoswitchable GFP-like protein with fluorescence excitation decoupled from switching. *Nat. Biotechnol.* **29**, 942–947 (2011). 10.1038/nbt.1952 21909082

[4] Habuchi, S. et al. Reversible single-molecule photoswitching in the GFP-like fluorescent protein Dronpa. *Proc. Natl. Acad. Sci. USA* **102**, 9511–9516 (2005). 10.1073/pnas.0500489102 PMC1157093 15972810

[5] Pennacchietti, F. et al. Fast reversibly photoswitching red fluorescent proteins for live-cell RESOLFT nanoscopy. *Nat. Methods* **15**, 601–604 (2018). 10.1038/s41592-018-0052-9 29988095

[6] Hell, S. W., Jakobs, S. & Kastrup, L. Imaging and writing at the nanoscale with focused visible light through saturable optical transitions. *Appl. Phys. A* **77**, 859–860 (2003).

[7] Hofmann, M., Eggeling, C., Jakobs, S. & Hell, S. W. Breaking the diffraction barrier in fluorescence microscopy at low light intensities by using reversibly photoswitchable proteins. *Proc. Natl. Acad. Sci. USA* **102**, 17565–17569 (2005). 10.1073/pnas.0506010102 PMC1308899 16314572

[8] Grotjohann, T. et al. Diffraction-unlimited all-optical imaging and writing with a photochromic GFP. *Nature* **478**, 204–208 (2011). 10.1038/nature10497 21909116

[9] Masullo, L. A. et al. Enhanced photon collection enables four-dimensional fluorescence nanoscopy of living systems. *Nat. Commun.* **9**, 3281 (2018). 10.1038/s41467-018-05799-w PMC6095837 30115928

[10] Boden, A. et al. Volumetric live cell imaging with three-dimensional parallelized RESOLFT microscopy. *Nat. Biotechnol.* **39**, 609–618 (2021). 10.1038/s41587-020-00779-2 33432197

[11] Casas Moreno, X. et al. Multi-foci parallelised RESOLFT nanoscopy in an extended field-of-view. *J. Microsc* **291**, 16–29 (2023). 10.1111/jmi.13157 36377300

[12] Prakash, M., Delbracio, M., Milanfar, P. & Jug, F. Interpretable unsupervised diversity denoising and artefact removal. In *Proc. 10th International Conference on Learning Representations* https://openreview.net/forum?id=DfMqlB0PXjM (OpenReview.net, 2022).

[13] Marín-Aguilera, G. et al. All-optical strategies to minimize photobleaching in reversibly switchable fluorescent proteins. *Nat. Commun* **16**, 10843 (2025). 10.1038/s41467-025-67009-8 PMC12672575 41326380

[14] Waldchen, S., Lehmann, J., Klein, T., van de Linde, S. & Sauer, M. Light-induced cell damage in live-cell super-resolution microscopy. *Sci. Rep.* **5**, 15348 (2015). 10.1038/srep15348 PMC4611486 26481189

[15] Krull, A., Buchholz, T. O. & Jug, F. Noise2Void - learning denoising from single noisy images. In *Proc.**2019 IEEE/CVF Conference on Computer Vision and Pattern Recognition (CVPR) 2124–213*2 https://openaccess.thecvf.com/content_CVPR_2019/html/Krull_Noise2Void_-_Learning_Denoising_From_Single_Noisy_Images_CVPR_2019_paper.html (IEEE, 2019).

[16] Qu, L. et al. Self-inspired learning for denoising live-cell super-resolution microscopy. *Nat. Methods* **21**, 1895–1908 (2024). 10.1038/s41592-024-02400-9 39261639

[17] Weigert, M. et al. Content-aware image restoration: pushing the limits of fluorescence microscopy. *Nat. Methods* **15**, 1090–1097 (2018). 10.1038/s41592-018-0216-7 30478326

[18] Ebrahimi, V. et al. Deep learning enables fast, gentle STED microscopy. *Commun. Biol.* **6**, 674 (2023). 10.1038/s42003-023-05054-z PMC10300082 37369761

[19] Priessner, M. et al. Content-aware frame interpolation (CAFI): deep learning-based temporal super-resolution for fast bioimaging. *Nat. Methods* **21**, 322–330 (2024). 10.1038/s41592-023-02138-w PMC10864186 38238557

[20] Cao, R., Divekar, N. S., Nunez, J. K., Upadhyayula, S. & Waller, L. Neural space-time model for dynamic multi-shot imaging. *Nat. Methods* **21**, 2336–2341 (2024). 10.1038/s41592-024-02417-0 PMC11621023 39317729

[21] Heine, J. et al. Adaptive-illumination STED nanoscopy. *Proc. Natl. Acad. Sci. USA* **114**, 9797–9802 (2017). 10.1073/pnas.1708304114 PMC5604029 28847959

[22] Dreier, J. et al. Smart scanning for low-illumination and fast RESOLFT nanoscopy in vivo. *Nat. Commun.* **10**, 556 (2019). 10.1038/s41467-019-08442-4 PMC6358637 30710076

[23] Fang, L. et al. Deep learning-based point-scanning super-resolution imaging. *Nat. Methods* **18**, 406–416 (2021). 10.1038/s41592-021-01080-z PMC8035334 33686300

[24] Zhang, Y. et al. Image super-resolution using very deep residual channel attention networks. In *Proc. European Conference on Computer Vision* (ECCV) 286–301 https://link.springer.com/chapter/10.1007/978-3-030-01234-2_18 (Springer, 2018)

[25] Soubannier, V., Rippstein, P., Kaufman, B. A., Shoubridge, E. A. & McBride, H. M. Reconstitution of mitochondria-derived vesicle formation demonstrates selective enrichment of oxidized cargo. *PLoS One* **7**, e52830 (2012). 10.1371/journal.pone.0052830 PMC3530470 23300790

[26] Hazan Ben-Menachem, R. et al. Mitochondrial-derived vesicles retain membrane potential and contain a functional ATP synthase. *EMBO Rep.* **24**, e56114 (2023). 10.15252/embr.202256114 PMC10157309 36929726

[27] Gustafsson, M. G. Surpassing the lateral resolution limit by a factor of two using structured illumination microscopy. *J. Microsc* **198**, 82–87 (2000). 10.1046/j.1365-2818.2000.00710.x 10810003

[28] Jin, L. H. et al. Deep learning enables structured illumination microscopy with low light levels and enhanced speed. *Nat. Commun.* **11**, 1934 (2020). 10.1038/s41467-020-15784-x PMC7176720 32321916

[29] Dertinger, T., Colyer, R., Iyer, G., Weiss, S. & Enderlein, J. Fast, background-free, 3D super-resolution optical fluctuation imaging (SOFI). *Proc. Natl. Acad. Sci. USA* **106**, 22287–22292 (2009). 10.1073/pnas.0907866106 PMC2799731 20018714

[30] Basak, S. et al. Super-resolution optical fluctuation imaging. *Nat. Photonics* **19**, 229–237 (2025).

[31] Zhao, W. S. et al. Enhanced detection of fluorescence fluctuations for high-throughput super-resolution imaging. *Nat. Photonics* **17**, 806–880 (2023).

[32] Tekpınar, M. et al. A lightweight deep learning framework for fast, real-time super-resolution fluctuation imaging. *Biophysical Rep (N Y)* **6**, 100279 (2026). 10.1016/j.bpr.2026.100279 PMC13524874 42562212

[33] Hell, S. W. & Wichmann, J. Breaking the Diffraction Resolution Limit by Stimulated-Emission - Stimulated-Emission-Depletion Fluorescence Microscopy. *Opt. Lett.* **19**, 780–782 (1994). 10.1364/ol.19.000780 19844443

[34] Minet, G. et al. Time-lapse RESOLFT nanoscopy empowered by deep learning. Zenodo, 10.5281/zenodo.22924956 (2026).42860407

[35] Minet, G. et al. Time-lapse RESOLFT nanoscopy empowered by deep learning. Zenodo, 10.5281/zenodo.22877255 (2026).42860407

[36] Casas Moreno, X., Al-Kadhimi, S., Alvelid, J., Bodén, A. & Testa, I. ImSwitch: Generalizing microscope control in Python. *J. Open Source Softw.* **6**, 3394 (2021).

