# Supplementary material for: Time-lapse RESOLFT nanoscopy empowered by deep learning
Source: Nat Commun. 2026 Oct 11;17:10657. doi: 10.1038/s41467-026-78580-z (PMC13654930; doi:10.1038/s41467-026-78580-z)
Supplement: Supplementary file 1 [file 41467_2026_78580_MOESM1_ESM.pdf]

Supplementary Information for

Time-lapse RESOLFT nanoscopy empowered by deep learning

Minet et al.

Corresponding Email: ilaria.testa@scilifelab.se, testa@kth.se

Supplementary Note 1: Effect of target quality and target pairing in supervised restoration

In standard supervised image-to-image training, the network is optimized to predict a target image by minimizing a pixel-wise loss such as L2 or L1. The optimal prediction will thus be the conditional mean or median of the target for a given input. When the target is experimental, it will inevitably contain some residual noise. In most experimental settings, the input and the target have been acquired with independent noise, meaning that the residual noise is not predictable from the input. Since the network cannot reproduce this fluctuating component consistently, it will instead converge toward an averaged solution that minimizes the expected error. This leads to a suppression of high-frequency fluctuations, too similar to noise, and appears as smoothing or blurring of fine features. This is why a small degradation of the ground-truth quality produces an increase in smoothing that we observed with all supervised trainings (Supplementary Figure S1). Additionally, it also explains why the same effect is expected for unsupervised approaches based on independent noisy pairs, such as Noise2Noise or Self-Inspired Noise2Noise, where the unpredictable part of the target tends to be averaged out.

However, this limitation does not apply in the same way to the supervised up-sampling task used for Fast-MoNaLISA, because the low-resolution input is generated directly from the target image by subsampling. The training pair is therefore perfectly matched: the target is not an independent noisy realization, but the exact high-resolution image from which the input was derived. Even if the target itself contains noise, that noise is intrinsically consistent with the input, so the mapping remains well defined and pixel-wise training does not induce the same averaging-driven smoothing effect as in supervised denoising. By contrast, if the target is replaced by another independently acquired fully sampled image, even with slightly better signal-to-noise ratio, the pair no longer shares the same pixel-level realization, and the prediction again produces a smoother prediction (see Supplementary Figure S11). This distinction is consistent with the broader super-resolution literature, where training is commonly performed on synthetically degraded pairs generated directly from the high-resolution target in order to preserve exact correspondence between input and output. However, the Fast-MoNaLISA benefits from the fact that this “synthetic degradation” is perfectly consistent with real fast acquisitions.

Supplementary Note 2: HDN and HDN^{sup} training

HDN is based on a Ladder Variational Autoencoder (LVAE), in which the network predicts a denoised signal $\hat{s}$ while learning a hierarchical latent representation of the image. Training minimizes

$$L = L_{\mathrm{rec}} + \beta L_{\mathrm{KL}},$$

where $L_{\mathrm{rec}}$is the reconstruction term and $L_{\mathrm{KL}}$is the Kullback–Leibler divergence. The KL term encourages the latent distribution inferred from each image to remain close to a simple prior, i.e. a standard normal distribution. In practice, this prevents the latent variables from storing arbitrary image-specific detail and instead promotes a structured representation of plausible clean solutions across scales, from coarse organization to fine texture.

In unsupervised HDN, the reconstruction term is derived from the experimentally calibrated noise model $p(x \mid s)$, which describes the probability of observing a noisy value x from an underlying clean signal s (see noise model section below). The network is trained by maximizing the likelihood of the observed input under this model:

$$L_{\mathrm{rec}} = -\log p(x \mid \hat{s}).$$

Thus, the model is not trained to reproduce the noisy image directly, but to predict a clean signal that could realistically have generated the measured observation through the known noise process. This is the key mechanism that enables denoising without clean targets.

In supervised HDN (HDN^{sup}), the same probabilistic framework is used, but the likelihood is evaluated against the target image y:

$$L_{\mathrm{rec}} = -\log p(y \mid \hat{s}).$$

In this case, the target acts as a stronger guide toward the desired clean solution, while the likelihood still defines a probabilistic measure of compatibility between the prediction and the target. Because the experimental noise model remains centered on the predicted signal, maximizing $p(y \mid \hat{s})$ still drives the prediction toward the target. In practice, this preserves the probabilistic character of the HDN framework while benefiting from supervised target information.

The parameter β controls the balance between reconstruction fidelity and latent-space regularization. Larger β values constrain the latent distributions more strongly, limiting the amount of image-specific information that can be encoded. This generally produces smoother predictions and increases the variability between sampled outputs, as the model retains more uncertainty about the underlying clean signal. Smaller β values allow the latent space to encode more specific details, leading to sharper and more

deterministic predictions, but with reduced diversity between samples. In the unsupervised setting, an intermediate value $\beta = 0.4 - 0.5$ is therefore needed to balance denoising strength, sharpness, and output diversity. In the supervised setting, because the target already constrains the solution strongly, a much smaller β is typically used, such as $\beta = 0.001$. See Supplementary Table 1 for exact β values used in this work.

At inference, multiple samples are generated and averaged to obtain the final prediction. In unsupervised HDN, this averaging stabilizes the estimate while integrating over several plausible restorations. Typically, 50 samples are used. In HDN^{sup}, the lower β leads to much smaller sample-to-sample variability, so fewer (~10) samples are sufficient in practice.

Noise Model Generation

The noise model $\boldsymbol{p}(\boldsymbol{x}|\boldsymbol{s})$ describes the conditional probability of observing a noisy value $\boldsymbol{x}$ for a given clean signal value s. This can be built experimentally from a paired dataset of clean-noisy images. Briefly, this is done by constructing a 2D histogram (e.g. 256x256 bins) with the clean signal values on the y-axis, and the noisy observations on the x-axis. Each row $\boldsymbol{j}$, once normalized, thus gives the probability $\boldsymbol{p}(\boldsymbol{x}|\boldsymbol{s_j})$. This histogram is then parametrized with a Gaussian Mixture Model (GMM). Although this step requires a paired dataset, it is important to note that it is only necessary to acquire it once for every imaging system and can then be used for any other HDN training, keeping the method essentially unsupervised.

Supplementary Note 3: Networks architecture

For HDN, we used a Ladder Variational Auto Encoder (LVAE) with 5 latent layers, 6 residual blocks per layer, and a latent space dimension of 32.

CARE was implemented as a U-Net with 4 downsampling layers, and 64 initial convolutional filters. For the UNet-RCAN used in the denoising task, we used a U-Net with 3 downsampling layers and 64 initial convolutional filters. For the RCAN we used 64 initial convolutional filters, 8 Residual Groups, and 12 Residual Channel Attention Blocks (RCAB). The usual skip connections of the U-Net were replaced by Channel Attention Blocks (CAB), as described in the original UNet-RCAN implementation.

For the upsampling task, we used the same U-Net and UNet-RCAN network architectures, without the CAB skip connections for the U-Net, but with an additional upsampling block at the end, consisting of a transposed convolution, an activation layer, and a final convolutional block. The detailed network parameters of UNet-RCAN for the upsampling method can be found in Supplementary Table 2.

Supplementary Note 4: Dataset acquisitions and preparation

This note provides additional details on the acquisition of paired, unpaired, and independent test datasets, as well as their preparation for network training and evaluation.

Paired datasets

To obtain a paired dataset with clean and noisy images, we fixed the cells, to avoid structural movements between pairs of images. The fixation limits the number of cycles of the rsFPs, meaning photobleaching will happen faster than in live cells. However, it is still possible to acquire high-quality RESOLFT images.

For the fixed vimentin dataset (U2OS Vim-rsEGFP2), the noisy images were first acquired at ~10-20 % ON-switching (~30W/cm2). Then, 5 images were acquired with ~80-90 % ON-switching (~300W/cm2). During this pseudo-timelapse, the signal will decrease rapidly. We used that signal decrease to evaluate HDN and HDNsup results at different SNR levels (Supplementary Fig. S2), and to evaluate the upsampling predictions for the different SNR levels (Supplementary Fig. S13). To create the best ground-truth possible, the first 3 images of the pseudo-timelapse were averaged.

For the fixed actin meshwork dataset (U2OS LifeAct-rsEGFP2), we used the same procedure, but the first image was acquired at ~50% ON-switching. The next 5 images were acquired at ~90% ON-switching and averaged to create a high-quality ground-truth and used to train HDNsup.

Unpaired datasets

Live unpaired datasets were used for both unsupervised denoising and upsampling training.

For unsupervised HDN denoising of mitochondria and HIV Gag-IM particles, low-ON-switching timelapses were acquired, and HDN was trained directly on these low SNR timelapses. Since each timelapse consists of many timepoints, only a few cells were needed to provide many noisy images.

For datasets used in the upsampling training, regular RESOLFT timelapses of 10–40 frames were acquired, the number of timepoints being adapted to each cell and structure to obtain sufficient SNR for the whole timelapse.

Independent test datasets

Two additional test datasets were acquired for HIV Gag-IM particles. The first one, used to evaluate the denoising, consists of images acquired in three conditions: low ON-switching, high ON-switching and enhanced confocal images. The second one, used to evaluate the upsampling, consists of only high ON-switching and enhanced confocal images. Because these datasets were still acquired on live cells, movement of the

structure prevents pixel-wise comparison. However, they could be used for visual validation of the prediction, as well as FRC comparison between the GT, the prediction, and the enhanced confocal.

Training dataset preparation

For each dataset, we first split the dataset into training, validation, and testing splits in a randomized way (See Supplementary Table 3 for full datasets details). We then extracted 64x64, 96x96 or 128x128 patches, and removed patches containing less information. This is done by normalizing the L2 norm of each patch to the maximum L2 norm of the whole training dataset and discarding the patches with a normalized L2 smaller than 0.1.

For the denoising tasks, these patches can be used directly. For the upsampling tasks, it is first required to generate the subsampled patches.

For the single-frame static training (Main Fig. 3b, Supplementary Fig. S9), we generated the 4 possible subsampled patches by simply selecting 1 pixel every 2 in each direction, with the 4 possible starting points: (x=0,y=0), (x=1,y=0), (x=0,y=1), (x=1,y=1). For S = 25 % we chose randomly 1 of the 4 patches. For S = 50 % we chose randomly 2 of the 4 patches and shuffled their order randomly. For S = 75 %, we chose randomly 3 of the 4 patches and shuffled their order randomly.

For the multi-frames temporal training (Main Fig. 4a), each acquired timelapse was first split into smaller timelapses of length N, before the patch extraction. N was always chosen as an odd number, so that each window contained a well-defined central frame T0, with (N-1)/2 preceding and following frames. Spatial patches were then extracted from each temporal window, resulting in input patches of size N×H×W, where the N timepoints were used as separate input channels, while the target is always the fully sampled central frame T0. For each frame in the temporal input, the S = 25% subsampled image was generated independently, exactly like in the single-frame patch preparation described above.

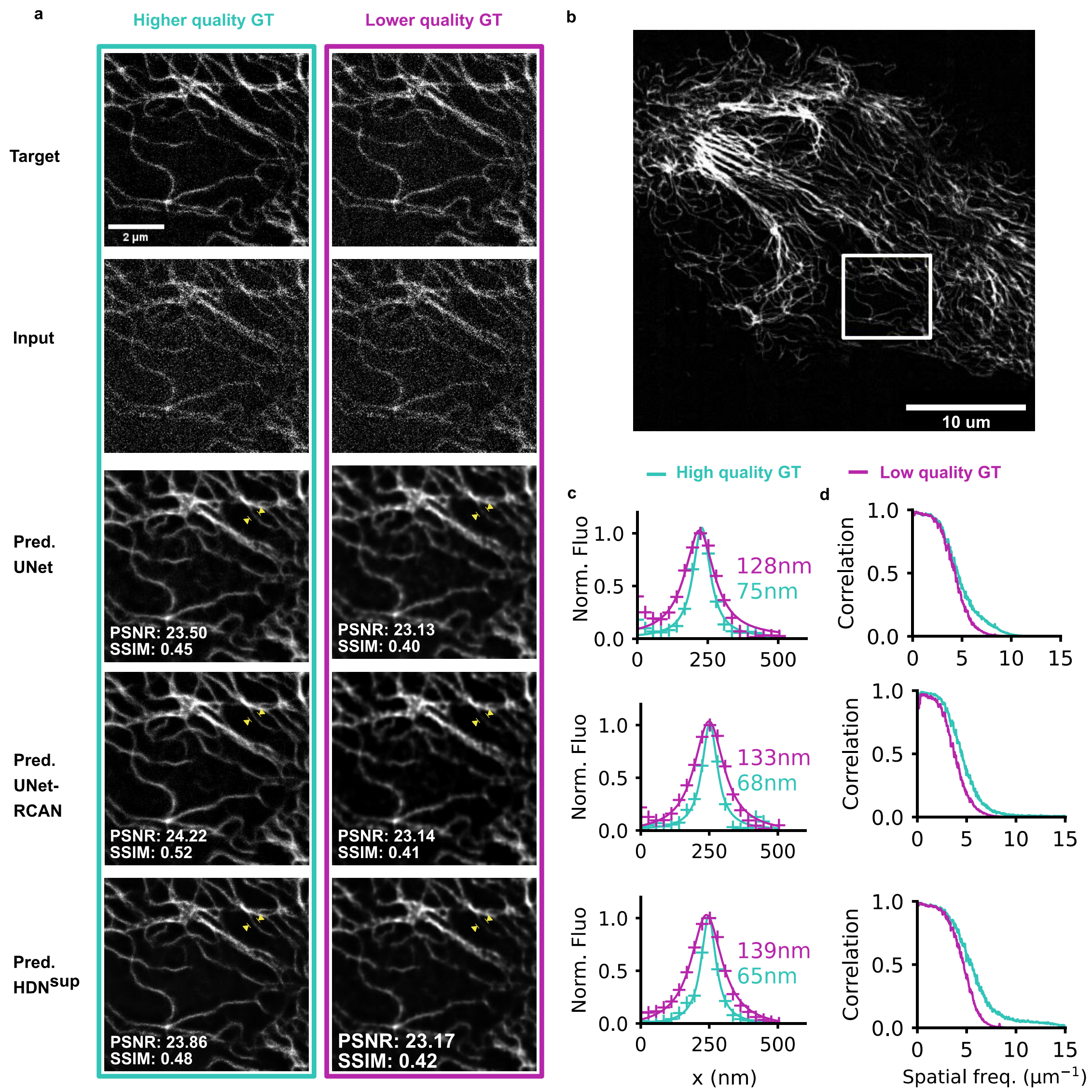

Supplementary Figure S1: Impact of the GT 's quality on supervised denoising

Prediction results for CARE, UNet-RCAN and HDN^{sup} for training two different GT quality on the Vimentin Dataset. **a** Images are zoom-in of ROI depicted in **b**. **b** Full FOV image. **c** Demonstration of smoothing effect when GT's quality lessens, with example line profile (orange arrows in **a**) and FRC curves.

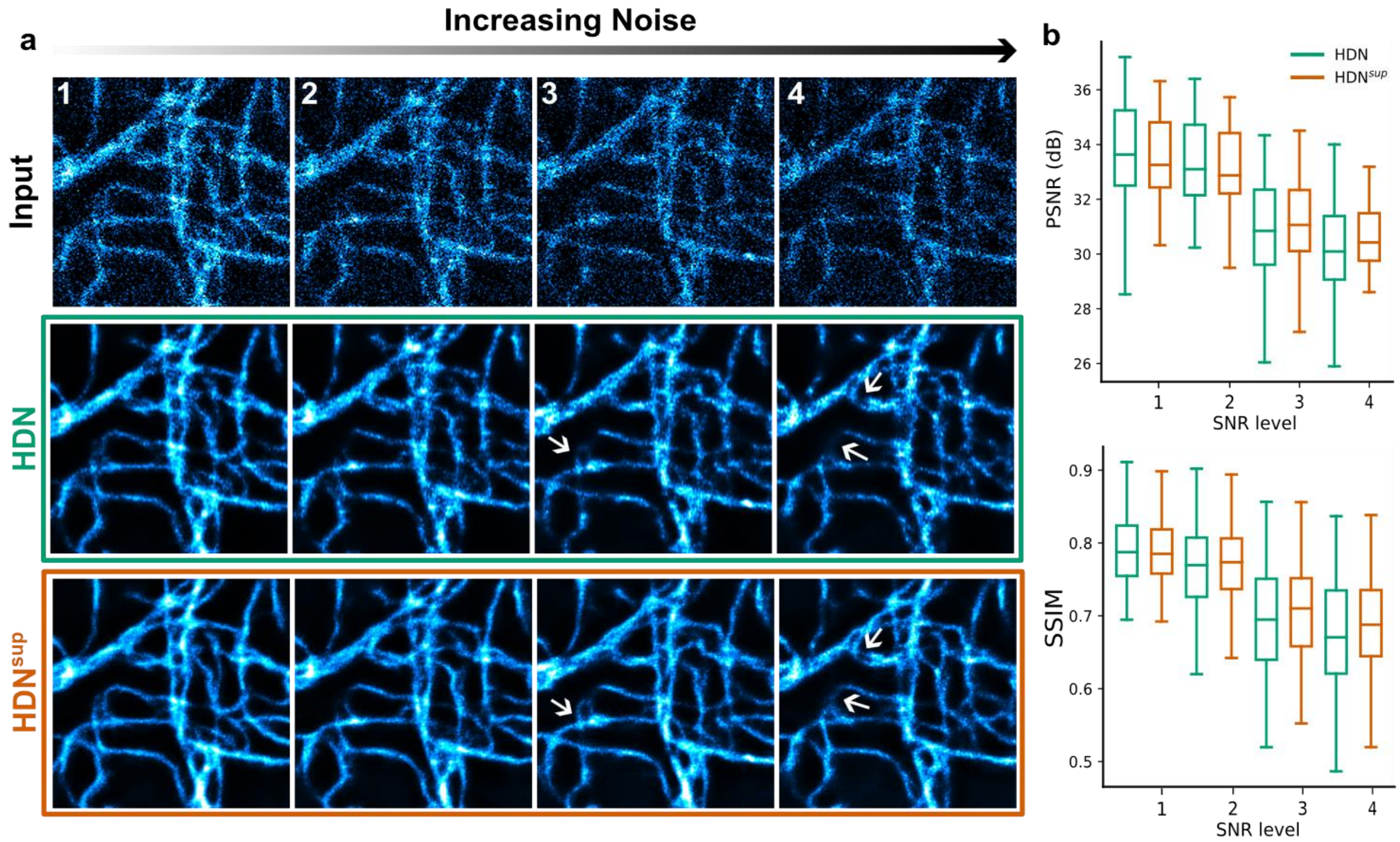

Supplementary Figure S2: HDN vs. HDN^{sup} for different SNR levels.

a Prediction example for HDN and HDN^{sup} for 4 different SNR input levels, on the fixed vimentin dataset. Different SNR have been obtained by letting the sample bleach over multiple acquisitions. Arrows show structures better recovered by HDN^{sup} for low SNR levels. **b** PSNR and SSIM over the whole test dataset (N=3 cells, cut in 600x600 images for more statistics). HDN^{sup} gives superior performances over HDN for lower SNR levels 3 and 4 for both metrics.

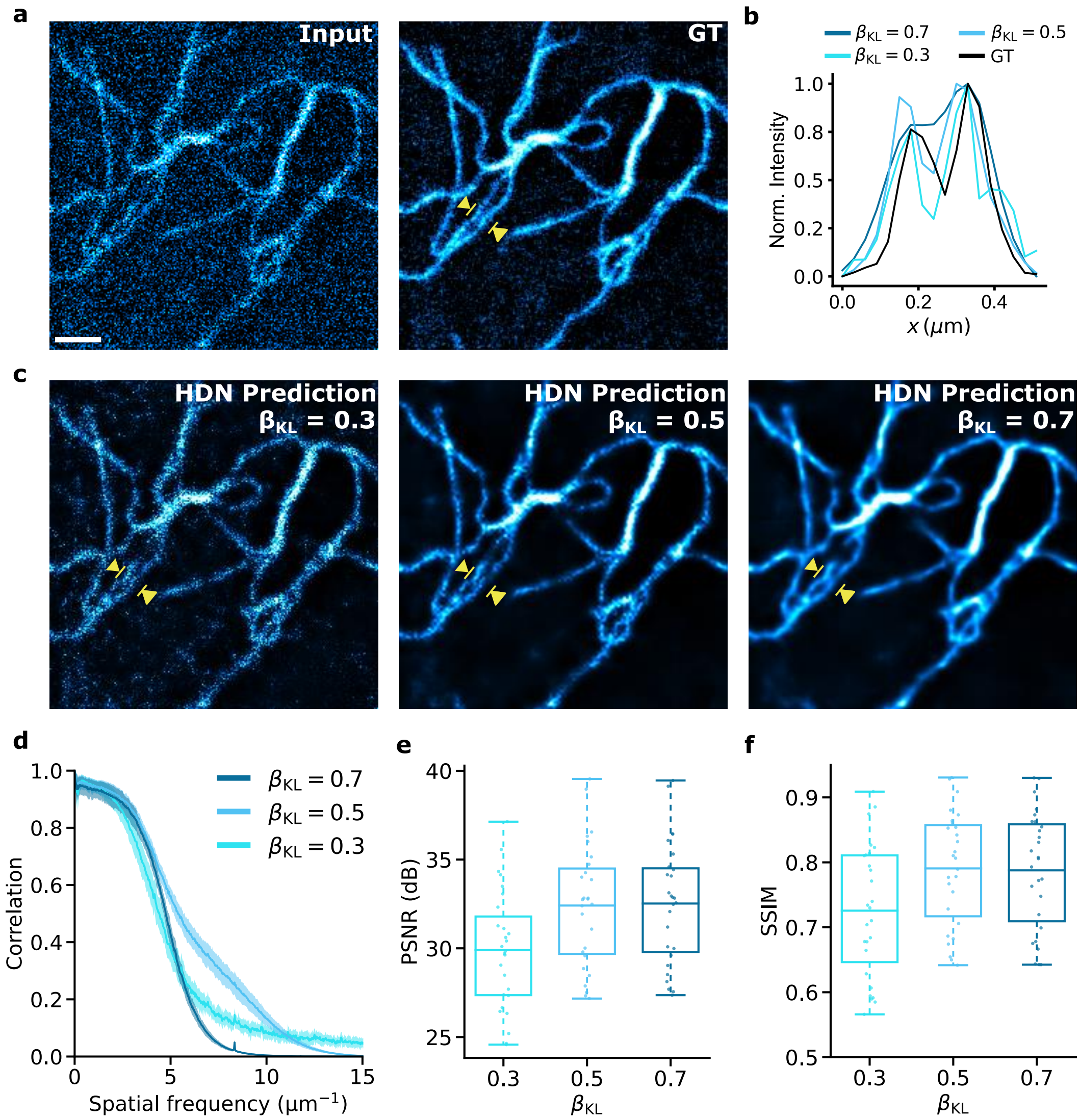

Supplementary Figure S3: Fine-tuning of the KL-loss weight β_{KL} for HDN training

The weight β_{KL} must be tuned to denoise enough without over smoothing. Here an example of this fine-tuning is shown for HDN training on the Vimentin dataset, with 3 weight values: too low (β_{KL} = 0.3), too high (β_{KL} = 0.7) and the selected value (β_{KL} = 0.5) which allows the best compromise between denoising and maintaining resolution. **a,c** GT, input and predictions results for the 3 β_{KL} values. Scale bar, 1um. **b** Line profile showing two close-by filaments in the GT (yellow arrows in **a**, **c**). Note that β_{KL} = 0.7 does not allow to distinguish the two structures. **d** 1FRC curves calculated over the whole training dataset (mean ± std, N=20 cells), showing a clear loss of resolution for β_{KL} = 0.7. **e** PSNR and SSIM over the whole training dataset (N=20 cells), showing that β_{KL} = 0.3 is too low for optimal SNR restoration.

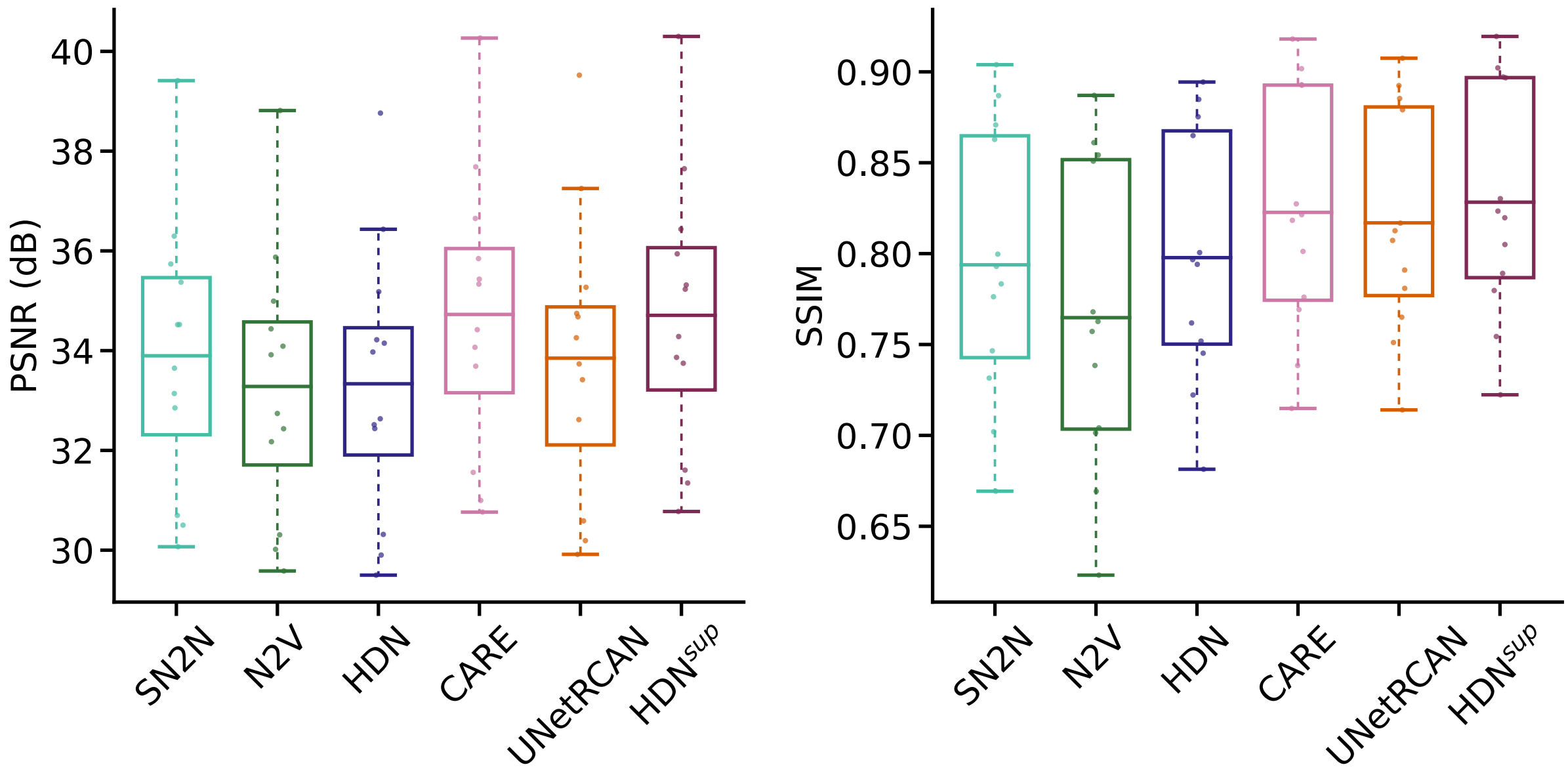

Supplementary Figure S4: PSNR and SSIM for all denoising methods (Vimentin)

PSNR and SSIM metrics for all denoising methods, calculated over the test dataset of fixed vimentin filaments (N=3 cells, cut in 600x600 images for more statistics).

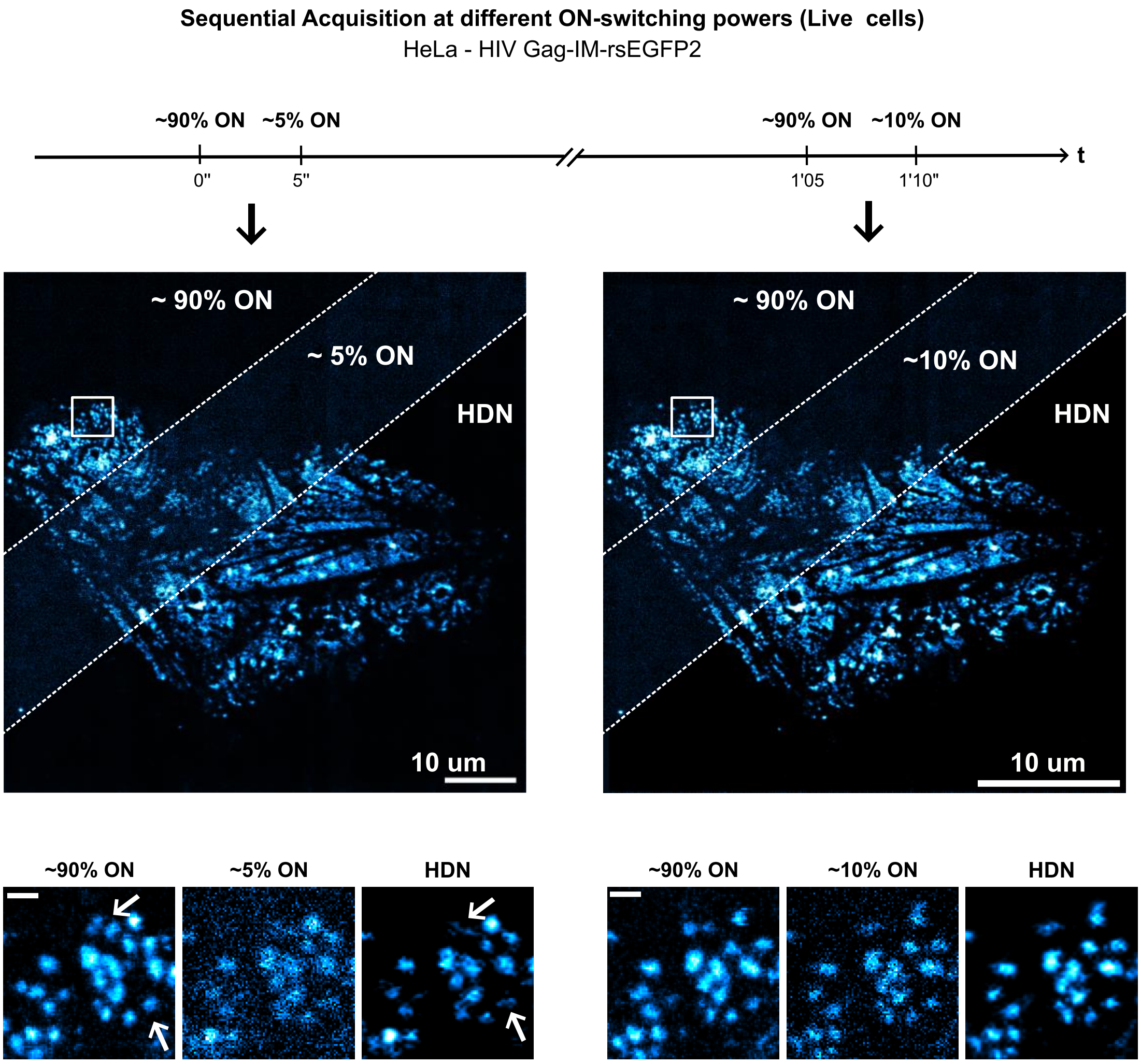

Supplementary Figure S5: ON-Switching level impact on HDN prediction

Example of how the ON-switching is fine-tuned for low-ON acquisitions. We acquired on live HeLa cells expresssing HIV Gag-IM-rsEGFP2, sequentially 4 images: ~5% ON-switching, ~90% ON-switching, ~10% ON-switching and ~90% ON-switching. The high SNR images acquired at ~90% ON-switching are used as visual control for each conditio, but can't be used for pixel-wise comparison because of the structure movement. In the zoom-in region of the ~5%, the prediction displays several artefacts (white arrows).

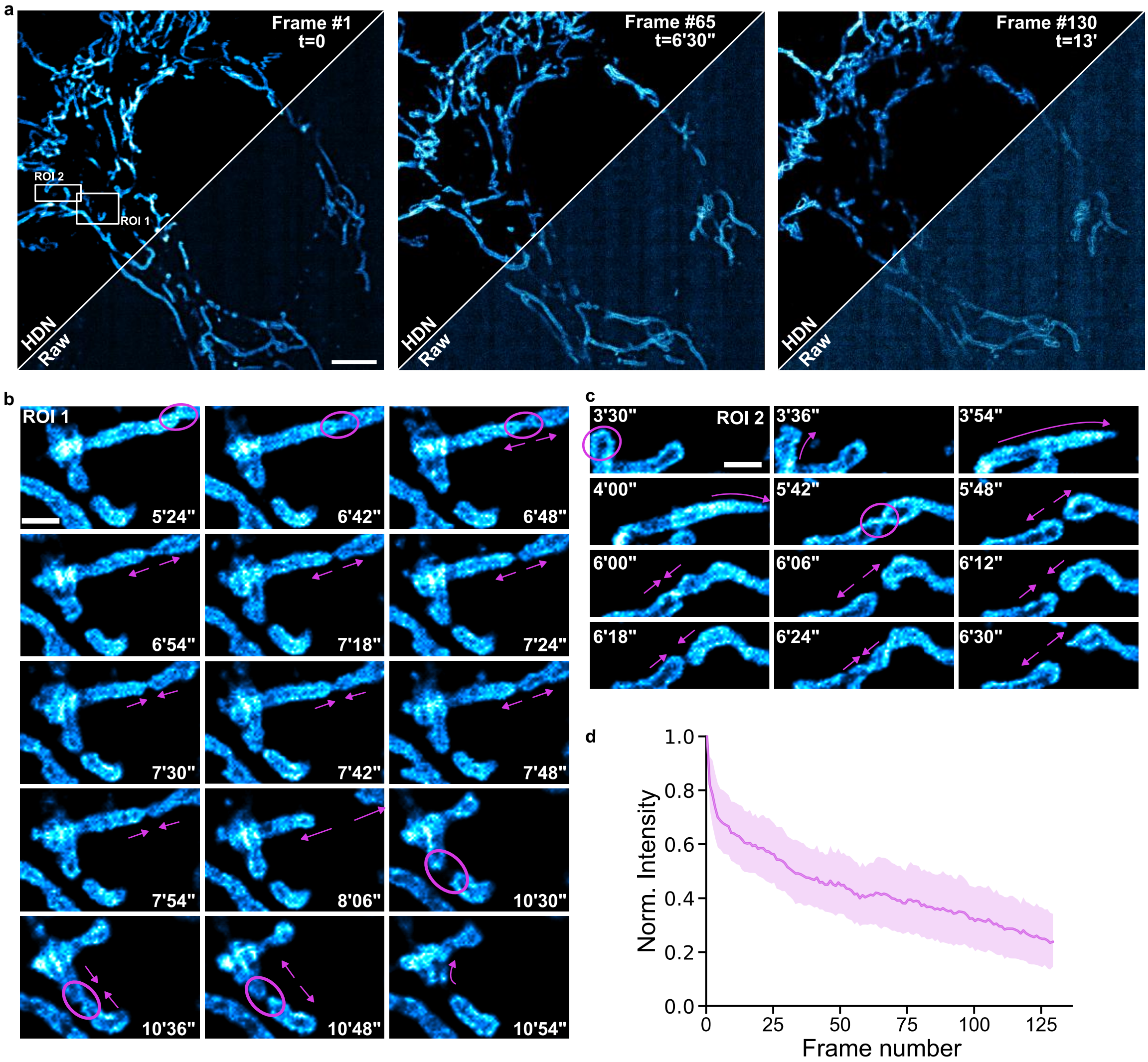

Supplementary Figure S6: Prolonged imaging OMP-rsEGFP2 in U2OS

MoNaLISA + HDN allows to image mitochondria in U2OS cells for 130 frames, ~4 times more than regular MoNaLISA. Frame rate: 6s. **a** Full FOV examples of raw and denoised for frame #1, #65 and #130. Scale bar, 5um. **b** Zoom-in over ROI1, showing first a mitochondria fission, followed by 2 back-and-forth contacts before definite separation happening (t=5’24” - t=8’06”), and another quick contact of 2 mitochondria between (t=10’30” - t=10’54”). Pink arrows highlight the movement of the mitochondria. Scale bar, 1um. **c** Zoom-in over ROI2, showing one mitochondrion first quickly extending, then later splitting into 2, and contacting again before definite fission. Scale bar, 1um. **d** Normalized fluorescence intensity over the 130 frames (on raw data, mean ± std, N=4 cells).

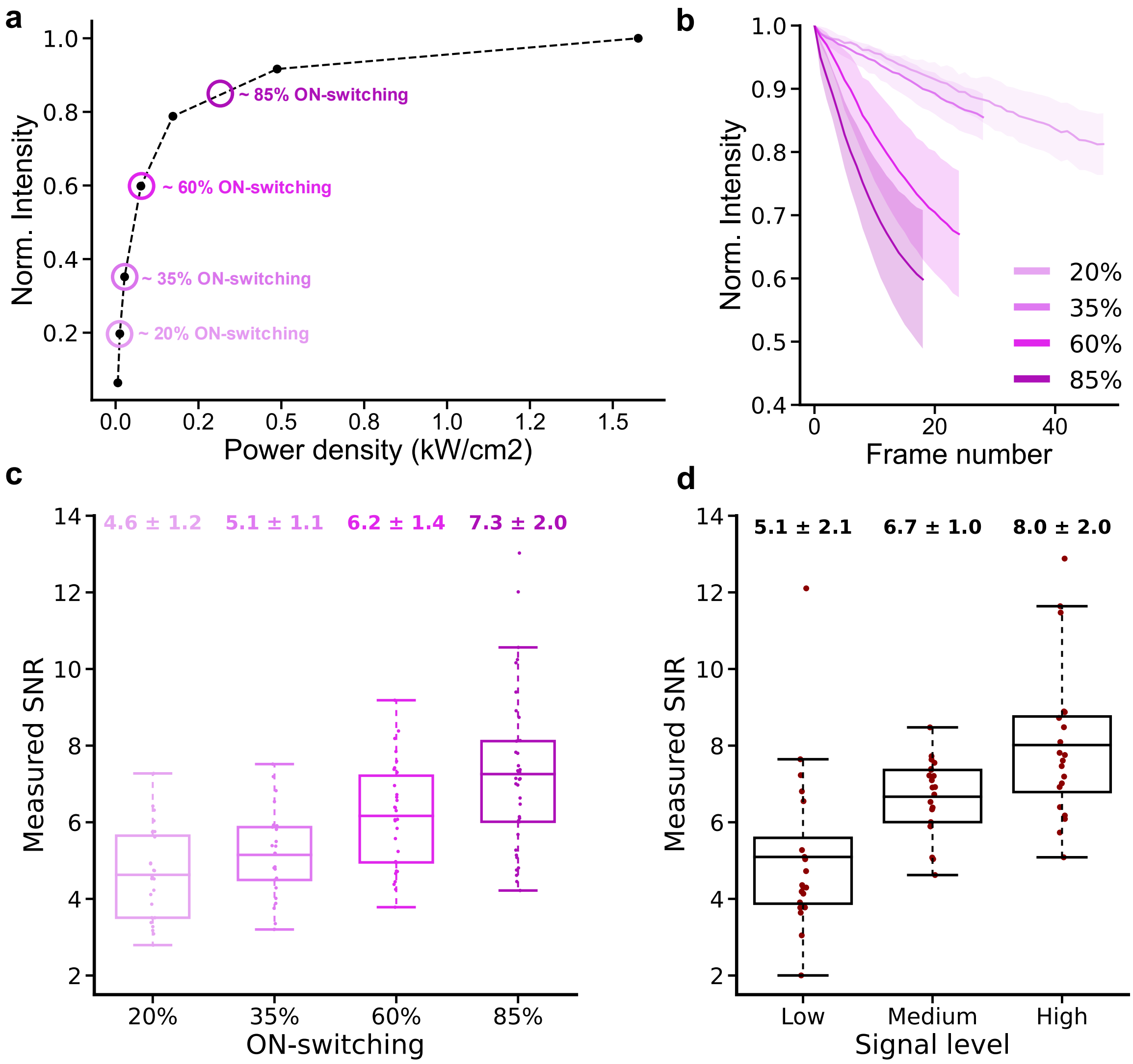

Supplementary Figure S7: ON-switching, bleaching and SNR on Vimentin-rsEGFP2.

a Experimentally obtained ON-switching curve. The ON-switching levels chosen for bleaching measurements are indicated with circles. **b** Bleaching rate depending on the ON-switching (mean ± std, N=5 cells for each ON-switching level). **c** Measured SNR for each ON-switching level, on live-cells, on the first frame of the timelapse. Mean ± std is indicated at the top of each box. SNR measurements are done from line profiles (see supplementary Figure 6), using N=25-30 filaments per ON-switching level. **d** Measured SNR on the fixed cells dataset, where the different signal levels (High, Medium, Low) have been obtained by letting the sample bleach, using N=20 filaments per signal level.

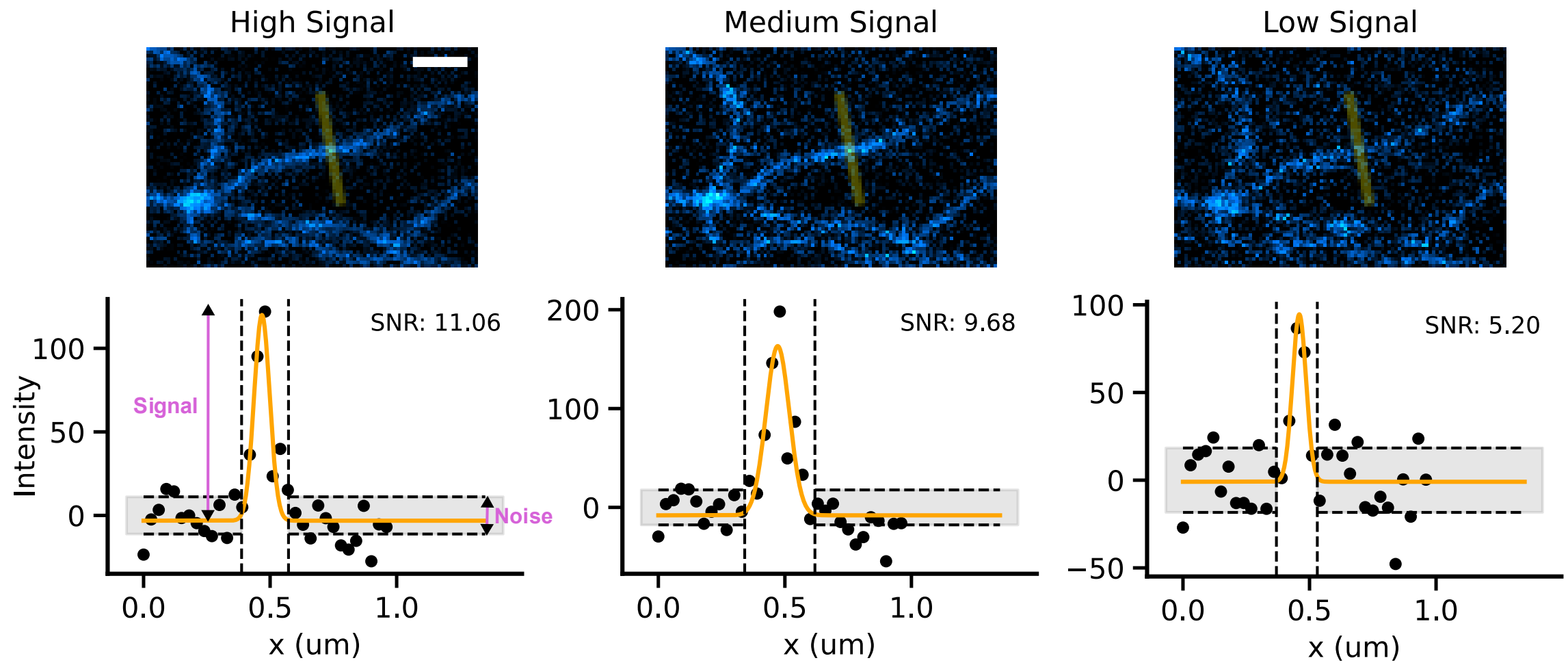

Supplementary Figure S8: Example of SNR calculation.

We calculate SNR as a ratio between the signal and the std of the background. For that, we use the line profiles of filaments. The signal is taken as the peak value of the fitted gaussian, and values outside of gaussian are considered as background. Here we show an example of such line profiles on the fixed Vimentin dataset for the 3 different signal levels, giving SNR values of 11.06 (High), 9.68 (Medium), and 5.20 (Low).

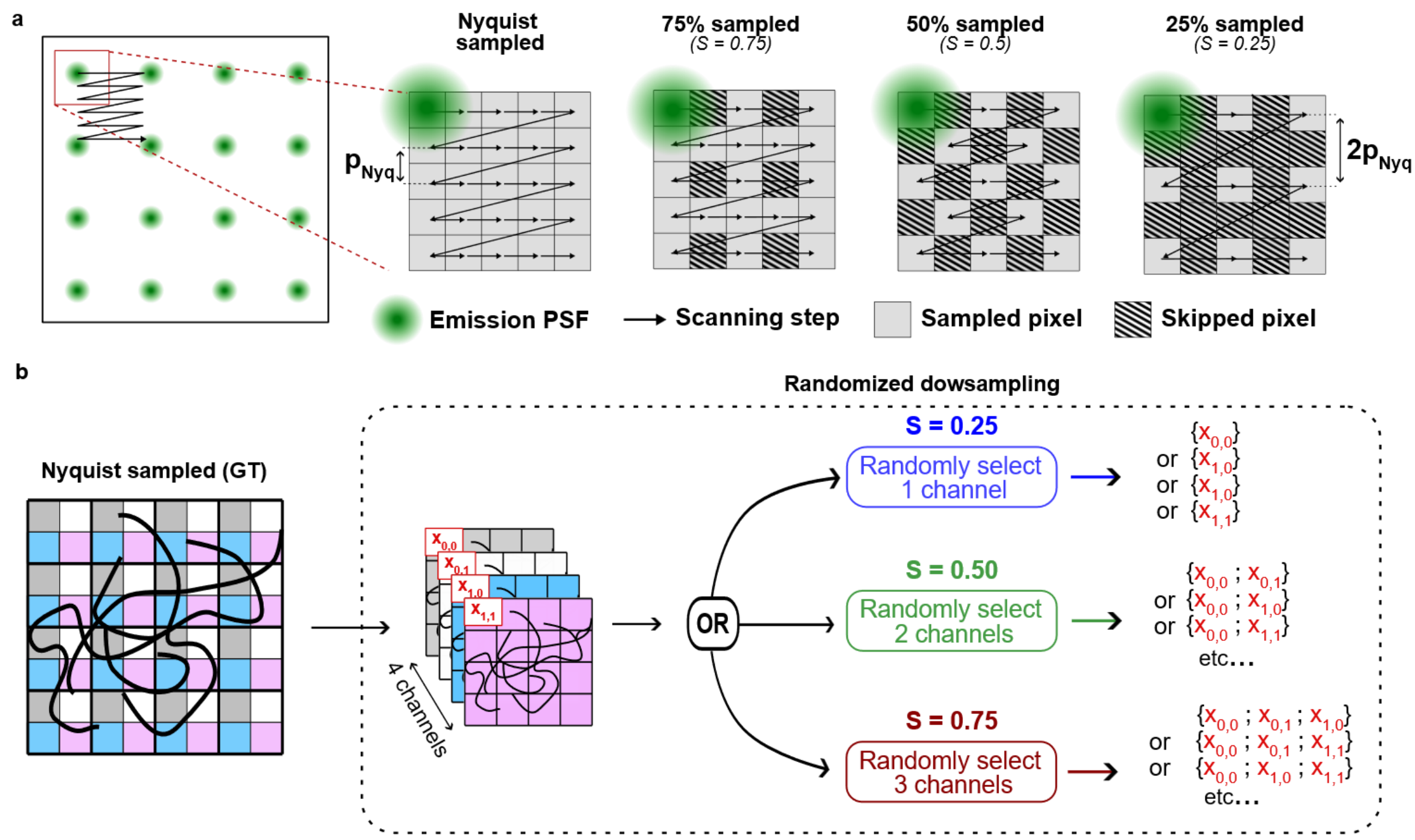

Supplementary Figure S9: Sub-sampling strategies and randomized downsampling

a In a parallelized system, we use a Raster Scan in between 2 emitting PSFs. A Nyquist sampling corresponds to a scan step of $\mathbf{p_{NYQ}}$**,** i.e half of the expected resolution, in both XY direction. Doubling the scan step to $\mathbf{2p_{NYQ}}$ in both directions will remove 75% of the information, creating an image sampled at only 25% (S=0.25). By skipping only a quarter or half of the scans steps, we can have intermediate sub-sampling images: 75% sampling (S=0.75) and 50% sampling (S=0.5). **b** Randomized downsampling strategy for S=0.25, S=0.5 and S=0.75. From a Nyquist sampled GT, we first create 4 downsampled images, each one corresponding to a doubled pixel size, but with a different subset of pixel. From this stack of 4 channels, we can select 1,2, or 3 depending on the desired sampling ratio S. For a given S, the channel selection is randomized.

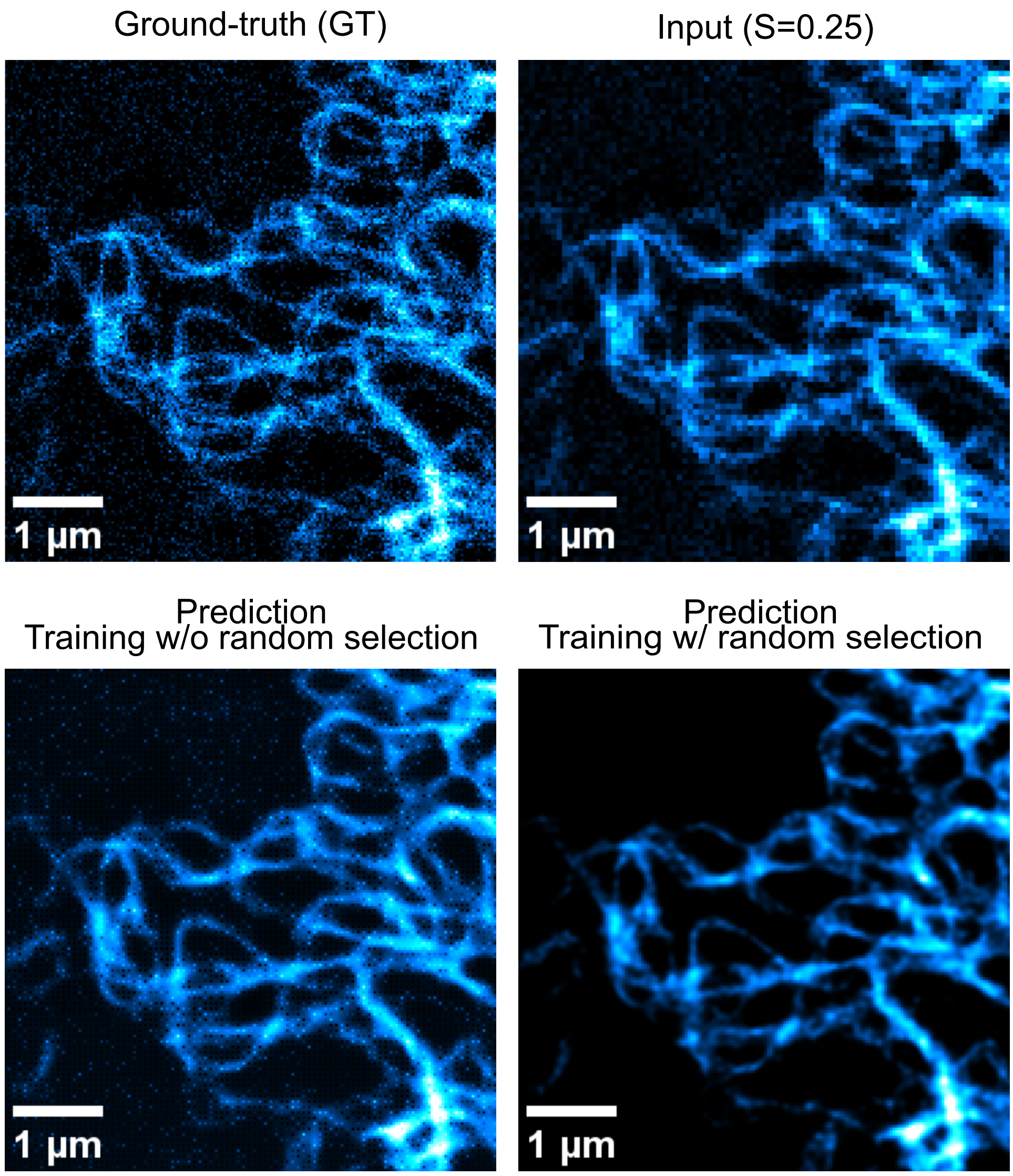

Supplementary Figure S10: Random vs deterministic pixel selection.

With a deterministic pixel selection during the training, the pixels in the input are always coming from the same positions from the GT. Therefore, it is easy for the network to learn that it should keep those exact pixels value to minimize the loss between prediction and GT. The GT's noise present in those pixels will thus be kept in the prediction. The other pixels that the network have to learn how to predict, however, will be necessarily more smoothed (the network cannot predict noise), creating this patterned noise effect. On the contrary, if the downsampling process does a random pixel selection, the network cannot know from where the input pixels are coming from. It must then converge towards a smooth output for all pixels.

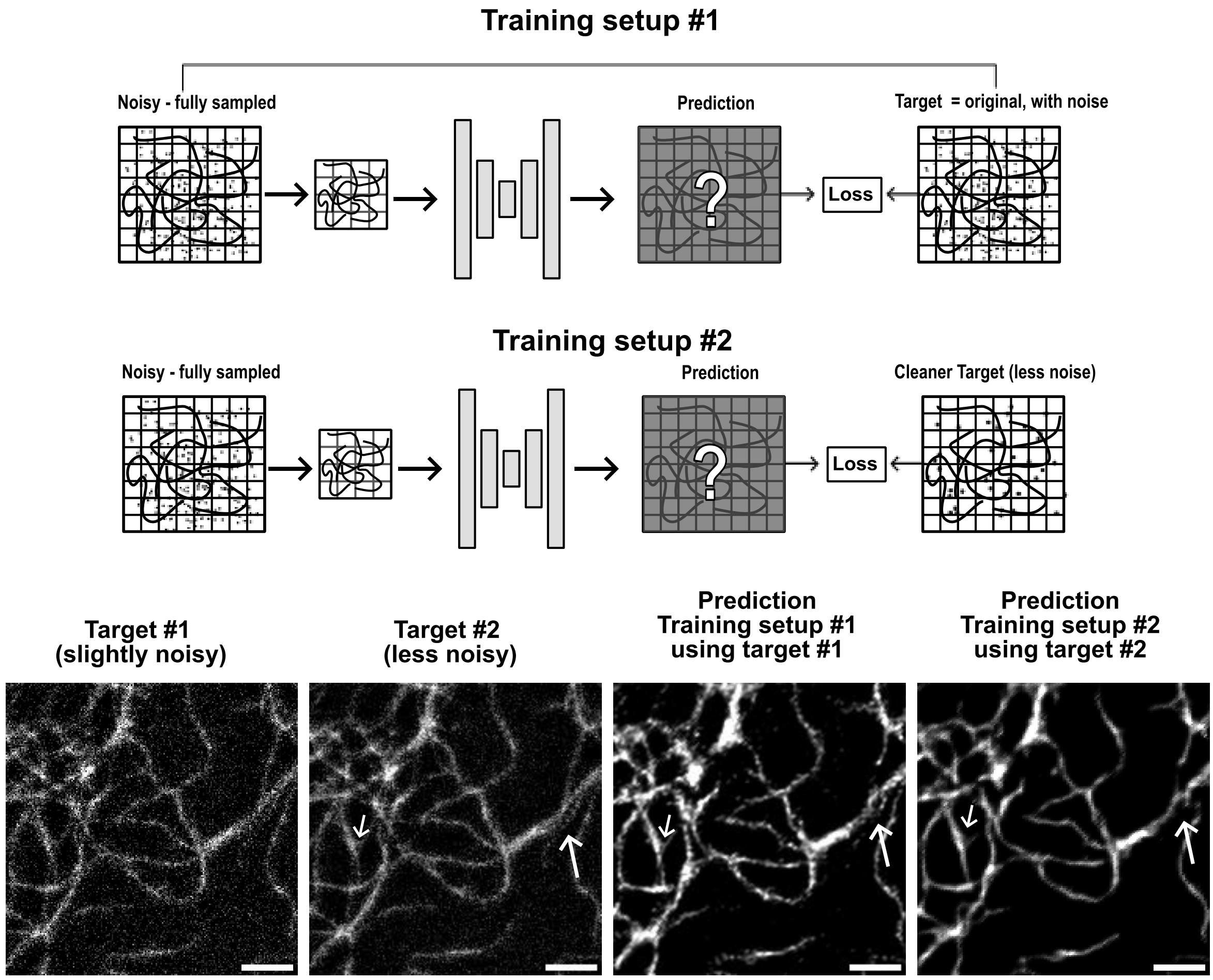

Supplementary Figure S11: Up-sampling task training strategies: original noisy GT vs cleaner GT

In the up-sampling task, the low-resolution input is generated directly from a fully-sampled image. When this fully-sampled image is noisy, and a cleaner fully-sampled image is available, two possible training setups are possible: keeping the original fully-sampled image as training target (training setup #1), or using the cleaner GT's as a target (training setup #2). In training setup #2, if the cleaner GT's still contain some noise, we observed a smoothing effect, which led us to keep training setup #1. White arrows show some details suppressed in prediction #2 due to the smoothing effect. Scale bar, 1 µm.

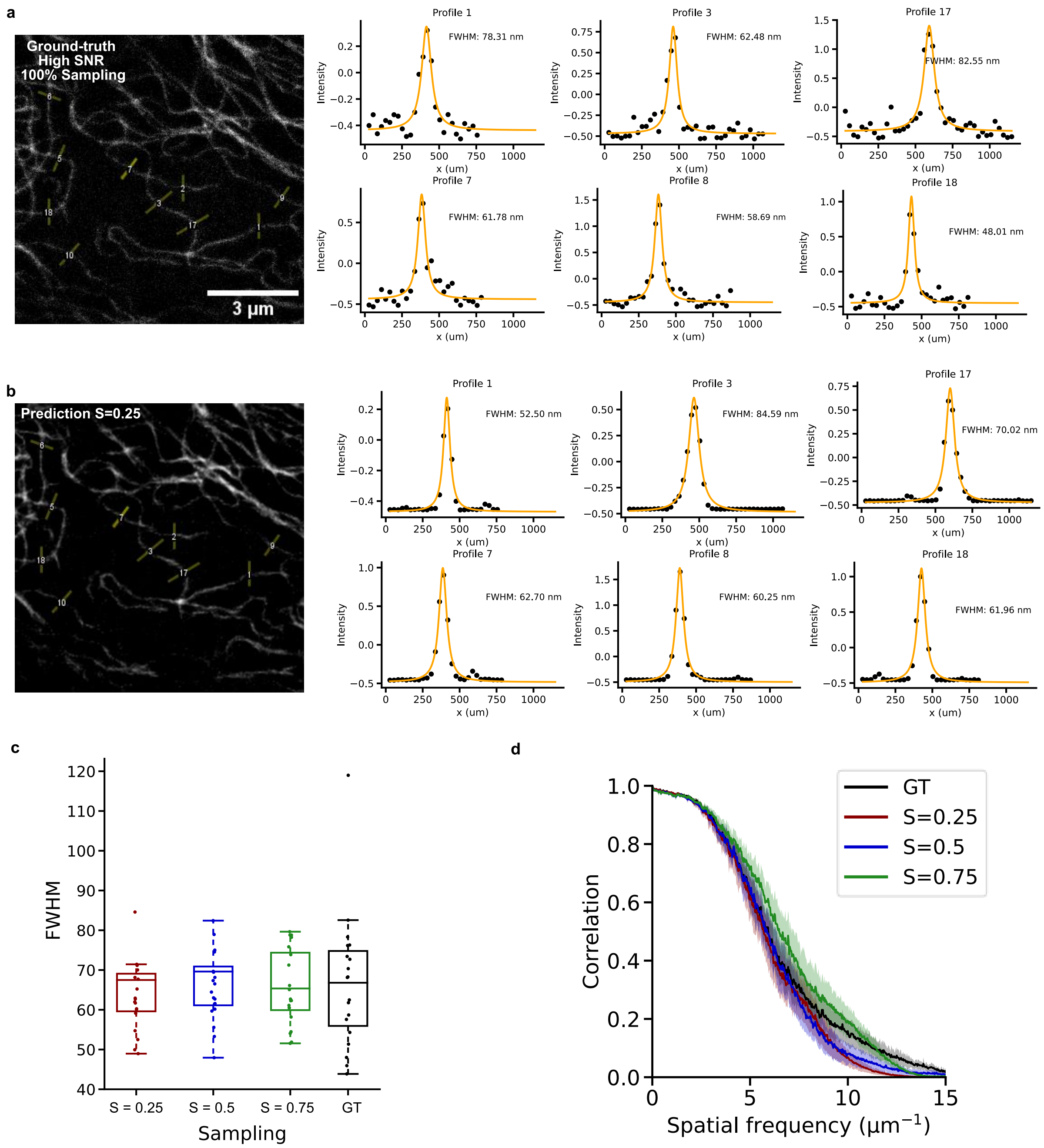

Supplementary Figure S12: Upsampling resolution characterization

To ensure that UNet-RCAN do not oversmooth the predictions in the up-sampling task, we performed line profiles FWHMs measurements and FRC calculation, for the GT, and the predictions for all 3 sampling ratios: S=0.25, S=0.50, S=0.75. **a,b** Zoom-in and example line profiles fits for the GT and S=0.25 prediction. **c** FWHM fits results for the GT and predictions. **d** FRC curves (mean +/- std, N=3 images).

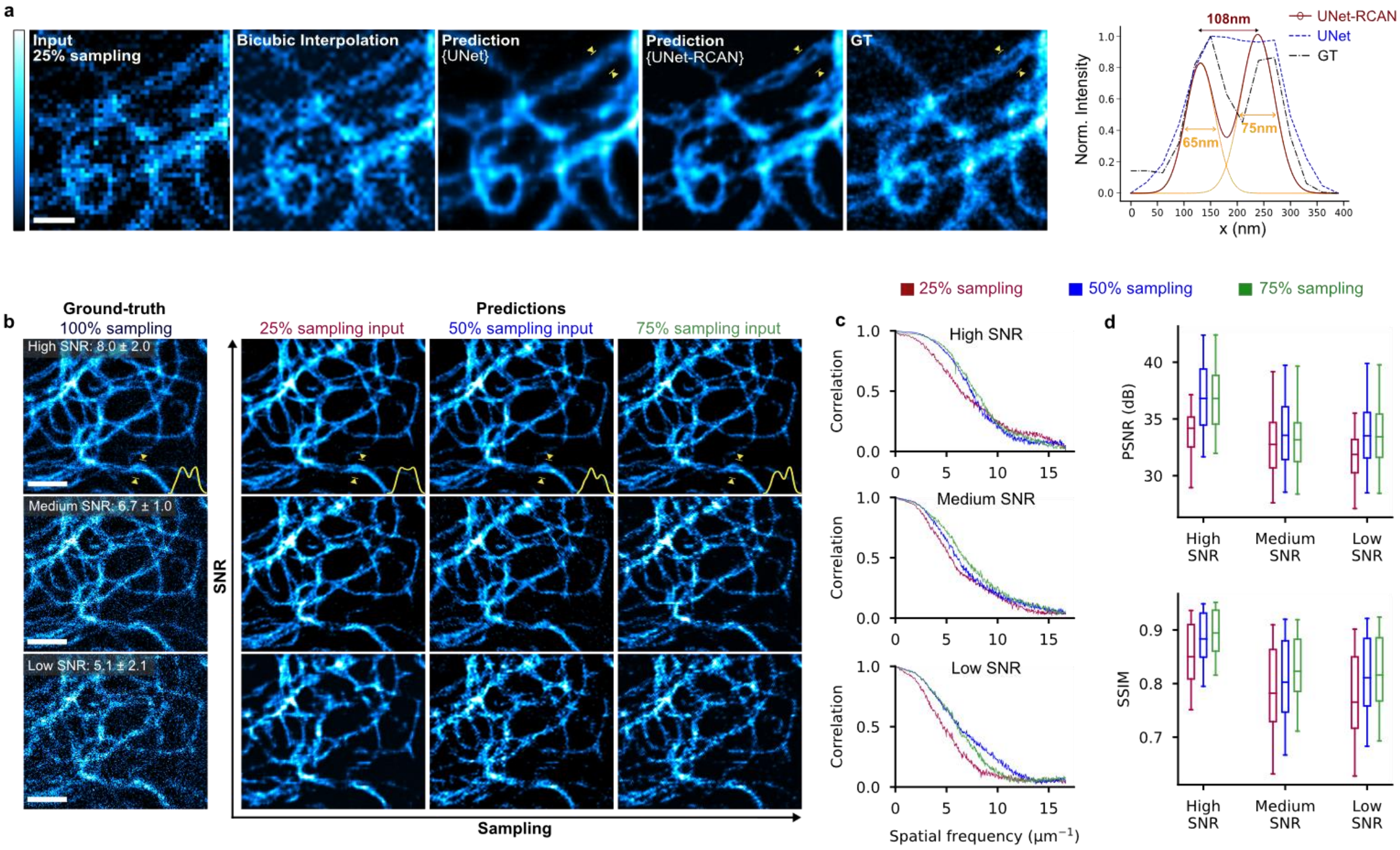

Supplementary Figure S13: Upsampling predictions for different sampling and SNR levels (Single-Frame training, Vimentin dataset)

a From left to right: 25 % sampling input, Bicubic Interpolation, UNet prediction, UNet-RCAN prediction, GT, and line profile comparison showing UNet-RCAN superior ability to resolve close-by filaments. **b** Prediction examples for 3 different SNR levels and 3 different sampling. Scale bar, 1 µm. **c** Relative FRC with GT for all SNR and sampling levels (N = 3 cells). **d** PSNR and SSIM calculation for all SNR and sampling levels. (N = 3 cells).

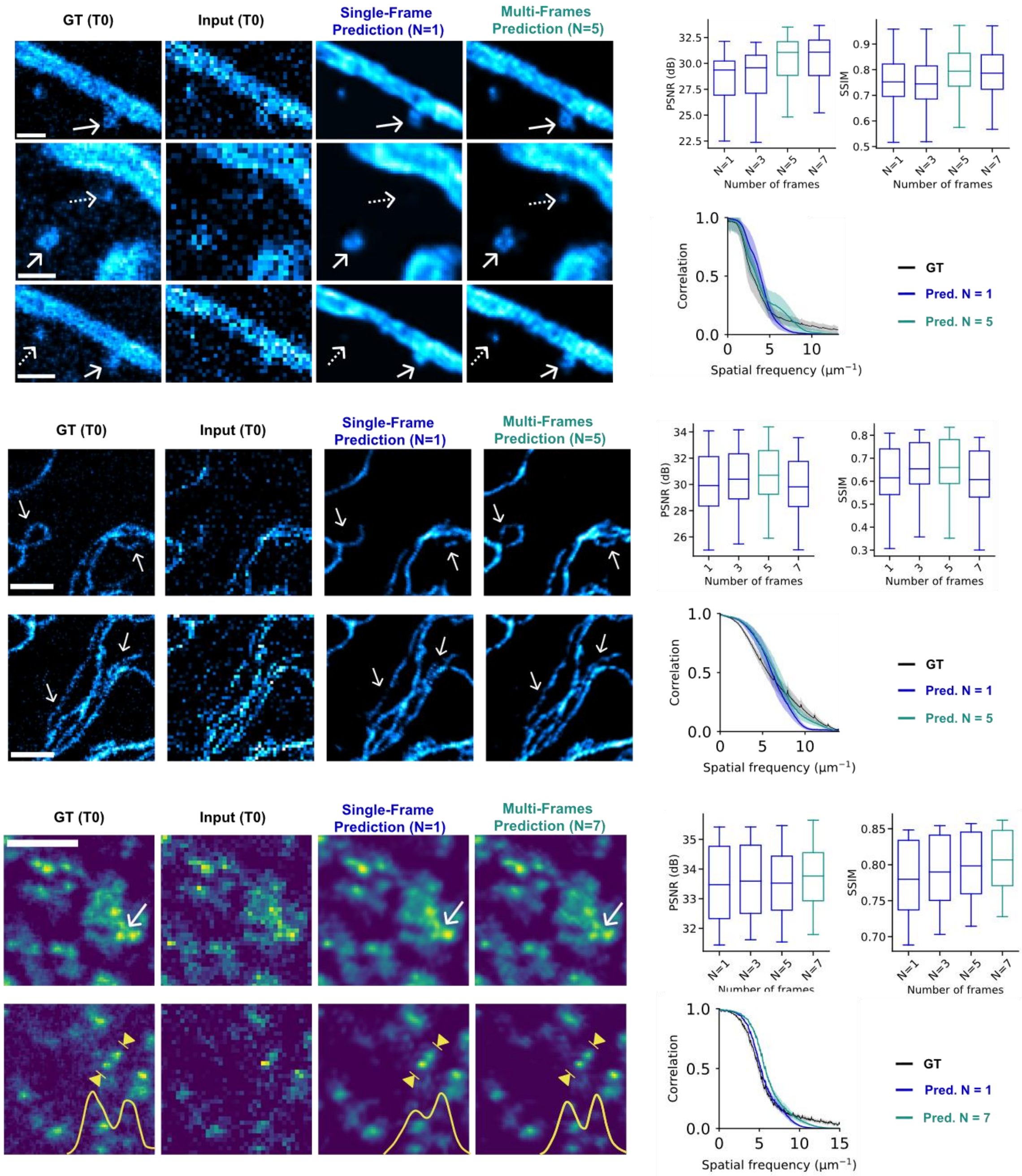

Supplementary Figure S14: Multi-Frame Training Results on Mitochondria, Vimentin and HIV Gag-IM

For each dataset, each row shows an example ROI of the fully-sampled (GT), the 25% sampled (input) and the predictions for single and multi-frames trainings. White arrows or inset line profiles in orange highlight improvements in the multi-frame predictions. For each dataset, PSNR and SSIM metrics for N=1,3,5,7 frames of the timelapse are shown, and the chosen number of frames is highlighted in cyan . Single-image FRC are shown for GT (fully-sampled), single and chosen multi-frames training.

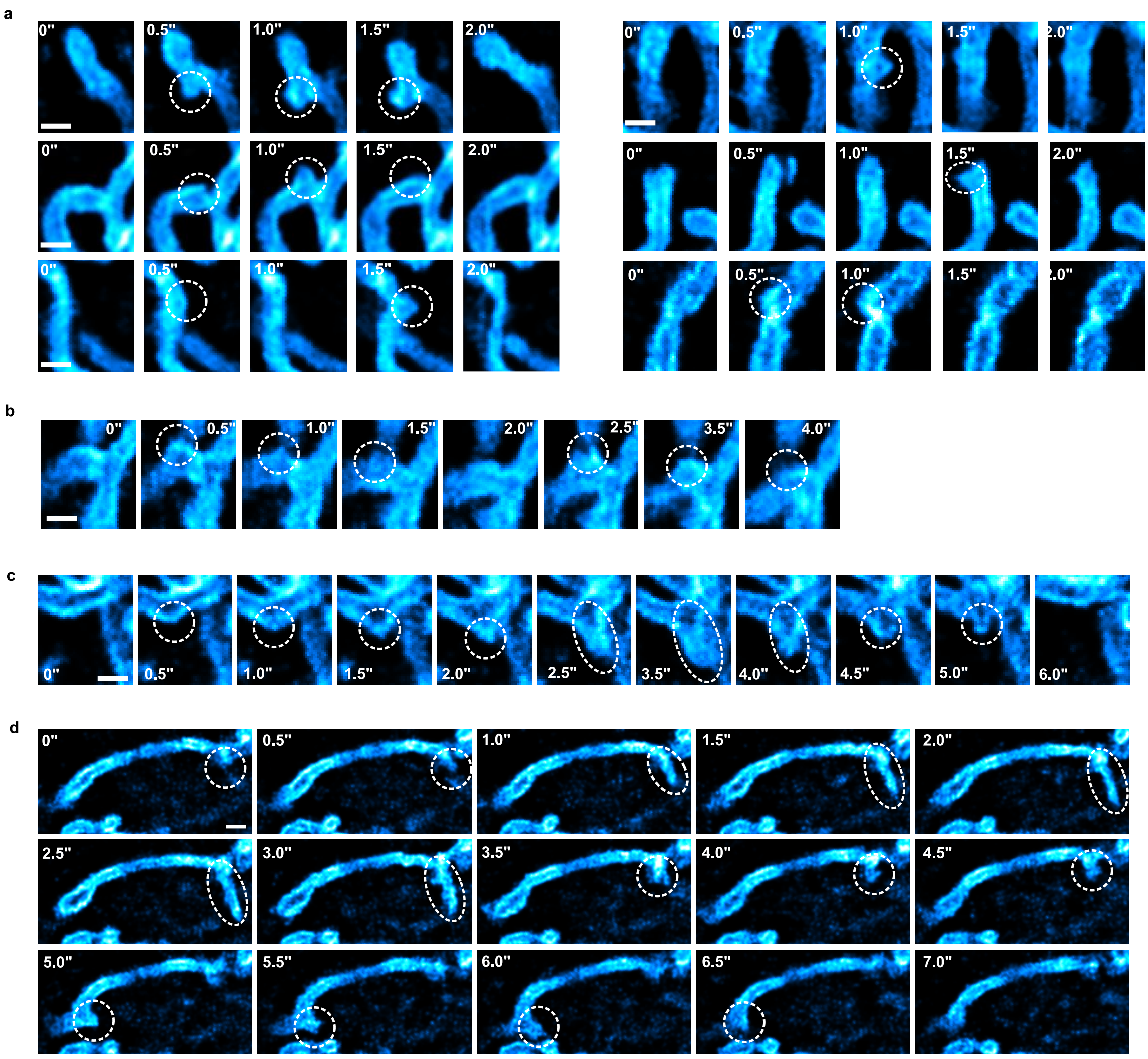

Supplementary Figure S15: Mitochondria fast protrusions observed with Fast-MoNaLISA

a 6 examples of fast and small mitochondria protrusion, elongating and retracting in 0.5-2”. **b** Example of 2 protrusions in a row. **c,d** Examples of longer, slower protrusions. All scale bars are 500nm. Frame rate is 2Hz.

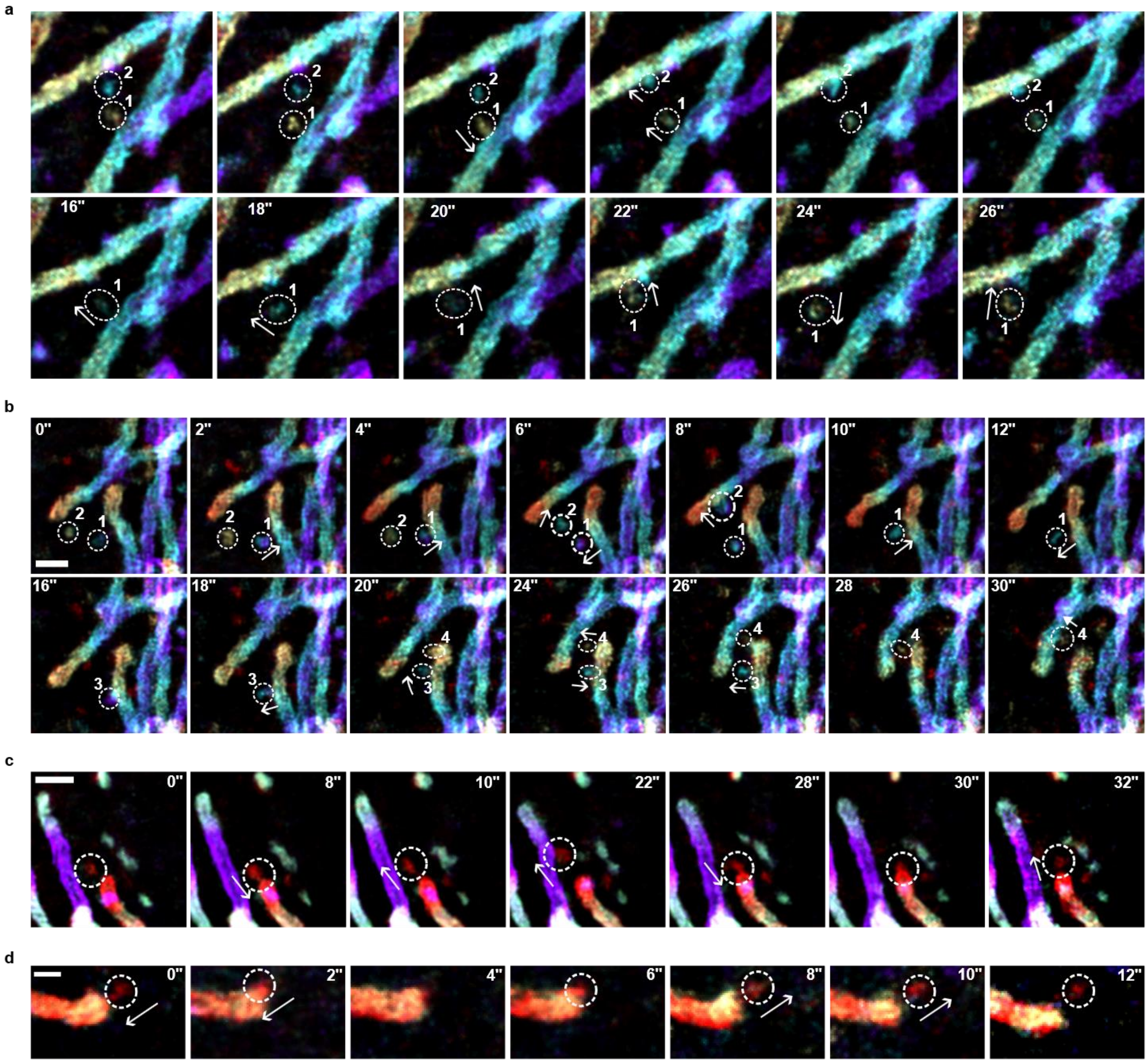

Supplementary Figure S16: MDV-mitochondria interactions examples

Multiple examples of Mitochondria Derived Vesicles (MDVs) budding out, fusing in, or touching multiple times mitochondria. Dotted circles shows the MDVs of interest, numbered when there are multiple ones in the same ROI. White arrows highlight the movement of the mitochondria. **a**, **b**, **c** scale bar 1um, **d** scale bar 500nm.

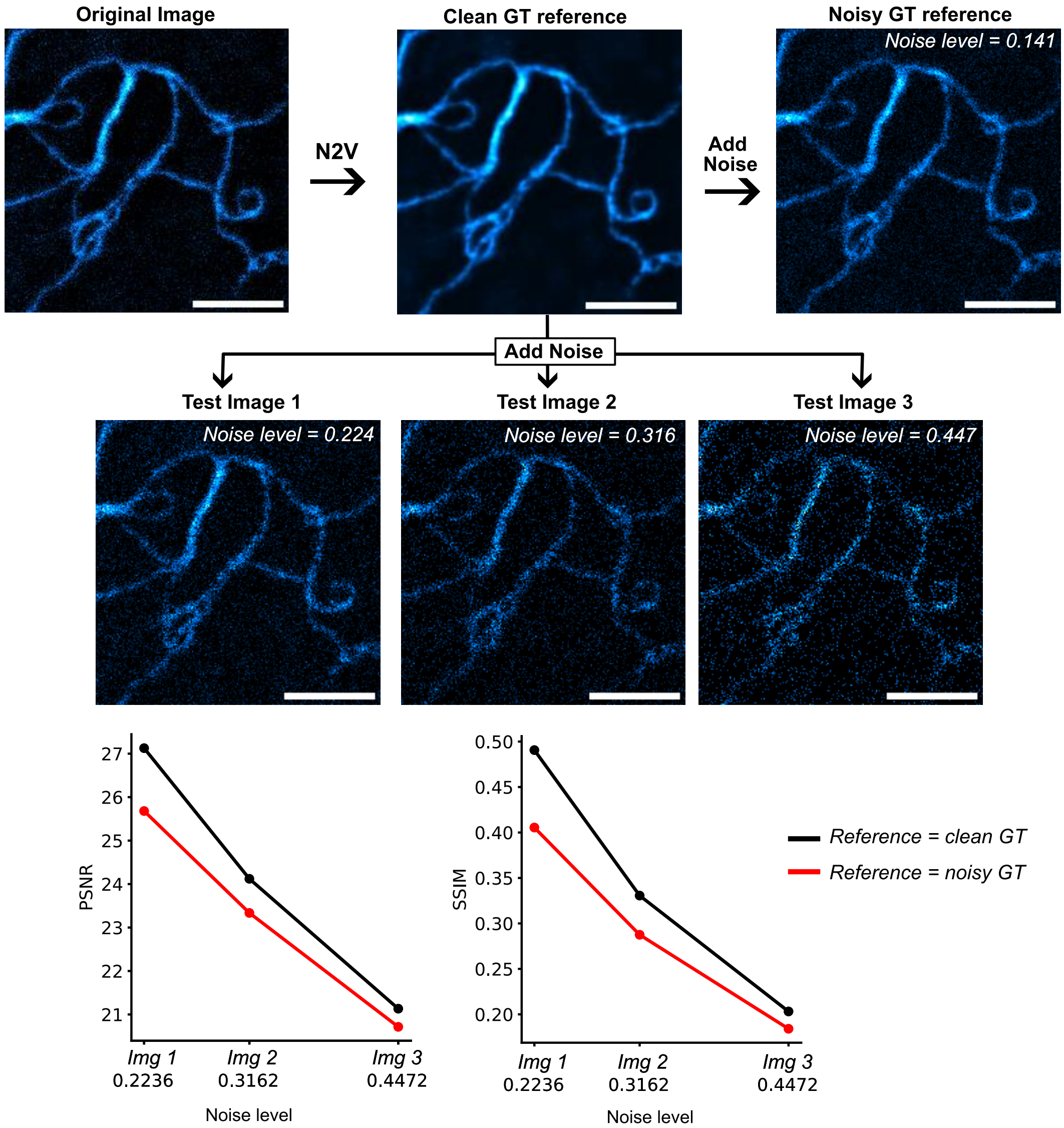

Supplementary Figure S17: Impact of GT's quality on PSNR/SSIM metrics

Starting from an experimental ground-truth image of U2OS Vim-rsEGFP2, we applied Noise2Void (N2V) denoising to obtain a clean reference image. A second reference image was then generated by adding a low level of Poisson noise, mimicking residual noise in an experimental ground truth. Three test images were generated by adding increasing levels of Poisson noise to the clean reference. PSNR and SSIM were computed for each test image using either the clean or the noisy reference. Noise levels were calculated as $1/\sqrt{P}$ where P is the peak maximum expected photons. We used $P = 50, 20, 10, 5$, giving noise levels of $\sim 0.141, \sim 0.224, \sim 0.316, \sim 0.447$, respectively. We demonstrate that metrics computed against a noisy ground truth provide a lower bound on the similarity to the underlying clean signal, and that relative comparisons between test images remain meaningful when the same reference is used, with noisier images consistently giving lower metric values. Scale bar, 2µm.

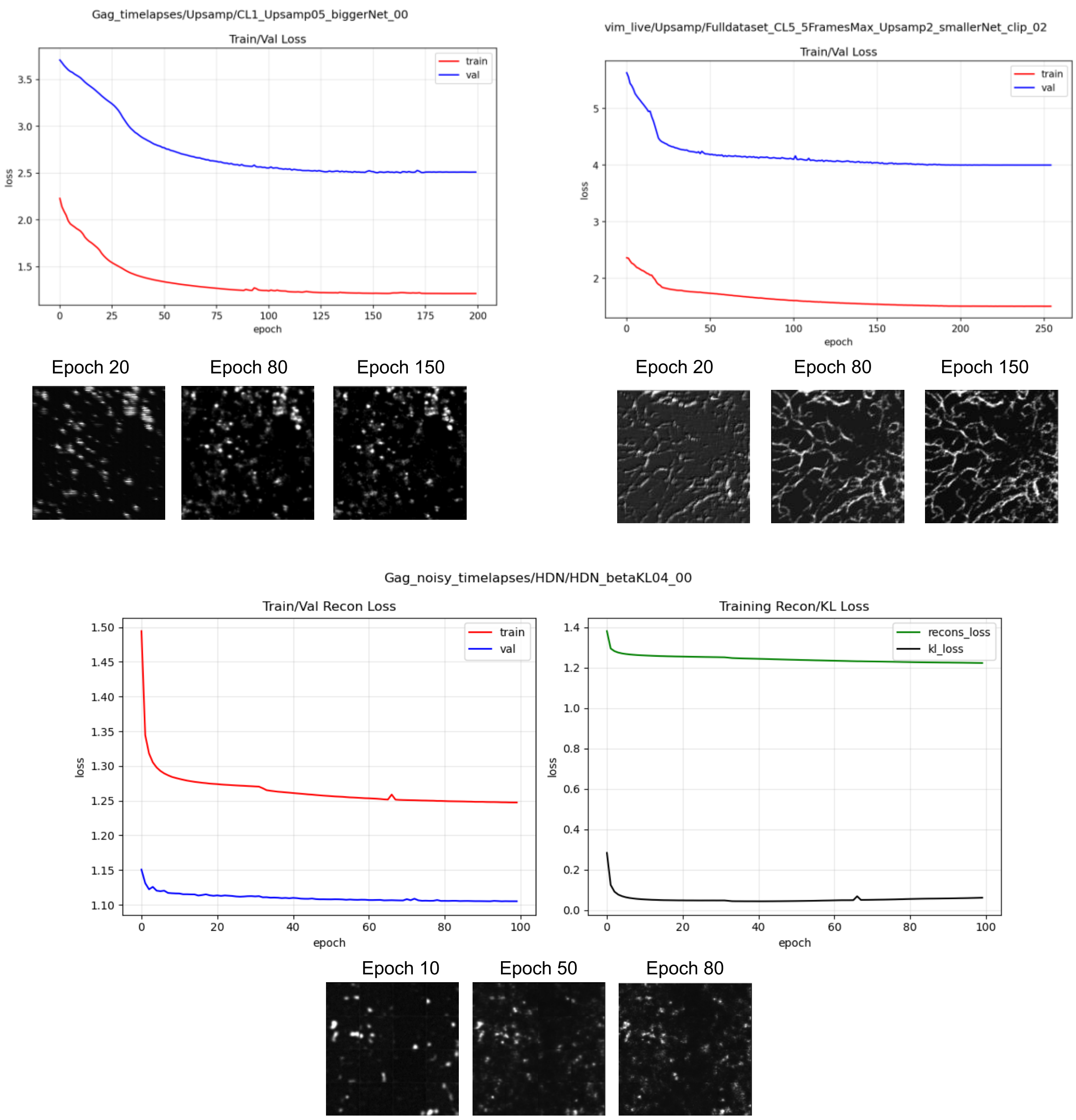

Supplementary Figure S18: Training curves examples

Training curves examples and validation images examples. Top left: HIV Gag-IM dataset, single frame upsampling training with S=0.5. Top right: live vimentin dataset, multi-frames training with N=5 frames. Bottom: HIV Gag-IM dataset, HDN denoising with reconstruction loss (training and validation) on the right, reconstruction and KL loss on the left (training only).

Dataset	Figure	Network Arch.	Patch size	# Patches	Learning Rate	Epochs	GPU Batch Size	Training Time***	β_{KL} (HDN only)
Fixed Vimentin (Multi SNR)	Main Fig.1 S1-4	UNet	128	1613	1×10^{-3}	100	32	0h31m	-
		UNet-RCAN	64	5467	1×10^{-4}	200	8	12h01m	-
		N2V	256	2419	4×10^{-4}	200	-	1h17m	-
		SN2N	128	7074	2×10^{-4}	200	-	0h28m	-
		HDN	64	5467	1×10^{-4}	200	8	17h32m	5×10^{-1}
		HDN ^{sup}	64	5467	1×10^{-4}	100	8	11h12m	1×10^{-2}
Fixed Actin	Main Fig.1	HDN ^{sup}	64	9953	1×10^{-4}	200	32	5h04	1×10^{-3}
Live Gag-IM (low ON)	Main Fig.1, S5	HDN	64	17171	1×10^{-5}	100	32	6h42m	4×10^{-1}
Live Mitochondria (low ON)	S5	HDN	64	10859	1×10^{-4}	100	32	4h04	2×10^{-1}

***Computed with NVIDIA RTX A4000. Note that experiments with GPU batch size of 8 are under-using GPU memory and could have been trained faster.

Supplementary Table 1: Training parameters and training time (Denoising)

Dataset Name	Figure	Network* params	Patch size	Learning Rate	Sampling (S) # frames (N)	# Training Patches	Epochs **	GPU Batch Size	Training Time***
Fixed Vimentin (Multi SNR)	Fig. 3, S10-13	UNet: depth 3 filters: 64<br><br>RCAN: filters: 64 rg = 8 rcab = 12 reduction =16	64	1×10^{-4}	S = 0.25 N = 1	4049	48	8	7h06min
					S = 0.50 N = 1		48	8	7h06mis
					S = 0.75 N = 1		85	8	11h29min
Live Vimentin (high ON)	Main Fig.4, S14	UNet: depth 3 filters: 64<br><br>RCAN: filters: 64 rg = 8 rcab = 8 reduction=12	96	1×10^{-4}	N=1 S = 0.25	1846	121	32	0h54min
					N=3 S = 0.25		298	32	2 h14min
					N=5 S = 0.25		254	32	1h54min
					N=7 S = 0.25		102	32	0h45min
Live Mitochondria (high ON)	Main Fig.4, S14-16	UNet: depth 3 filters: 64<br><br>RCAN: filters: 64 rg = 8 rcab = 12 reduction =16	96	5×10^{-5}	N=1 S = 0.25	9378	181	8	13h02m
					N=3 S = 0.25	7896	442	8	6h07min
					N=5 S = 0.25	5922	500	8	16h55min
					N=7 S = 0.25	3948	500	8	11h17min
Live Gag-IM (high ON)	Fig. 3, S14	UNet: depth 3 filters: 64<br><br>RCAN: filters: 64 rg = 8 rcab = 12 reduction =16	96	1×10^{-5}	N=1 S = 0.25	24343	200	32	24h18m
					N=1 S = 0.50	24343	190	32	23h27min
					N=3 S = 0.25	20964	200	32	20h50min
					N=5 S = 0.25	17614	200	32	17h35min
					N=7 S = 0.25	12505	78	32	4h50min

* Filters = initial number of convolutional filters. Rg = number of Residual Groups, RCAB = number of Residual Channel Attention Blocks and reduction= filters reduction ratio in CAB.
** Stopped when validation stagnates, automatically or manually, or when reached max number of epochs allowed.
*** Computed with NVIDIA RTX A4000. Note that experiments with GPU batch size of 8 are under-using GPU memory and could have been trained faster.

Supplementary Table 2: Training parameters and training time (Upsampling)

Dataset Name	Cell Type	Label	Fixed Or Live	Pixel Size nm	Image Size Ny*Nx px	Number of samples	Number of Timepoints (min-max)*	Total Number of images**	# train cells	# val cells	# test cells
Vimentin Fixed (Multi SNR)	U2OS	Vim-rsEGFP2	Fixed	30	1200x1200	27	-	27	22	1	3
Vimentin Live (high ON)	U2OS	Vim-rsEGFP2	Live	35	1000x1000	42	6-25	890	26	8	8
Fixed Actin	U2OS	LifeAct-rsEGFP2	Fixed	30	1200x1200	-	-	36	29	2	5
Live Gag-IM (high ON)	HeLa	HIV Gag-IM-rsEGFP2	Live	30	1200x1200	14	15-20		12	1	1
Live Gag-IM (low ON)	HeLa	HIV Gag-IM-rsEGFP2	Live	30	1200x1200	8	31-50	338	6	1	1
Live Mitochondria (high ON)	U2OS	rsEGFP2-Omp25	Live	34	336x1238	38	19-40	1013	26	4	8
Live Mitochondria (low ON)	U2OS	rsEGFP2-Omp25	Live	30	1300x1300	14	60-150	1524	18	3	3
**Test dataset** Eval Gag-IM Denoising	HeLa	HIV Gag-IM-rsEGFP2	Live	30	1200x1200	8	-	8	-	-	8
**Test dataset** Eval Gag-IM Upsampling	HeLa	HIV Gag-IM-rsEGFP2	Live	30	1200x1200	17	-	17	-	-	17

* Number of timepoints can vary, as the timelapse length is adapted to each sample's photobleaching rate.

** Total combined number of 2D images counting all the timepoints for all the samples.

Supplementary Table 3: Training and tests datasets description
