# Supplementary material for: Time-lapse RESOLFT nanoscopy empowered by deep learning
Source: Nat Commun. 2026 Oct 11;17:10657. doi: 10.1038/s41467-026-78580-z (PMC13654930; doi:10.1038/s41467-026-78580-z)
Supplement: Supplementary file 2 [file 41467_2026_78580_MOESM2_ESM.pdf]

Description of Additional Supplementary Files

Supplementary Movie 1: Prolonged mitochondrial imaging Timelapse of 130 frames, 6sec frame rate, denoised with HDN (unsupervised), of U20Scells expressing OMPrsEGFP2. Zoomed in region shows multiple fissions and multiple“back-and-forth contacts”, highlighted by white arrows.

Supplementary Movie 2: Timelapse of actin meshwork dynamics Timelapse of 50 frames, 5sec frame rate, denoised with HDNsup of U20S cells expressing LifeAct-rsEGFP2. Zoomed in region shows that we can follow the actin meshwork dynamics for ~50 frames.

Supplementary Movie 3: Mitochondria motion artefacts example in regular MoNaLISA Timelapse acquired with regular MoNaLISA, on a reduced FOV to achieve 1Hz frame rate, in U20S cells expressing OMP-rsEGFP2. Motion artefacts are highlighted by white circles.

Supplementary Movie 4: Mitochondria fast dynamics observed with 2D FastMoNaLISA Fast-MoNaLISA timelapse (2Hz, full FOV) of U20S cells expressing OMP-rsEGFP2. First zoomed-in region highlights very small and fast protrusions elongating and retracting. Second zoomed-in region shows other varieties of events observed.

Supplementary Movie 5: Fast volumetric imaging and MDV budding-out and budding-in Volumetric rendering of a Fast-MoNaLISA volumetric acquisition of U20S cells expressing OMP-rsEGFP2. Zoom-in region shows 2 Mitochondria Derived Vesicles (MDVs) budding-out and fusing with another mitochondrion.
