# Supplementary material for: Time-lapse RESOLFT nanoscopy empowered by deep learning
Source: Nat Commun. 2026 Oct 11;17:10657. doi: 10.1038/s41467-026-78580-z (PMC13654930; doi:10.1038/s41467-026-78580-z)
Supplement: Supplementary file 4 [file 41467_2026_78580_MOESM9_ESM.pdf]

nature portfolio

Peer Review File

Time-lapse RESOLFT nanoscopy empowered by deep learning

Corresponding Author: Professor Ilaria Testa

This file contains all reviewer reports in order by version, followed by all author rebuttals in order by version.

Version 0:

Reviewer comments:

Reviewer #1

(Remarks to the Author)
Minet et al. report deep learning based denoising methods for RESOLFT imaging, low-power nanoscopy suitable for live-cell imaging. Multiple approaches are demonstrated to achieve this goal, including supervised/unsupervised denoising, down/upsampling and multiframe training. The authors clearly show that their methods could benefit fast long-term RESOLFT imaging. Although not presented, it seems to be straightforward to apply this method to other live-cell super-resolution imaging techniques. Below are minor comments which would be greatly helpful to strengthen the manuscript.

1. The manuscript would benefit from improved structural clarity. The authors present two or three deep learning models addressing different tasks, denoising and up-sampling, but the use of distinct architectures for each somewhat reduces the methodological coherence and overall impact of the work. For example, the HDN model is chosen over the U-Net RCAN for denoising due to the smoothing artifacts observed in the latter. However, the same U-Net RCAN is employed in the up-sampling section without further discussion of these smoothing effects. Although the two tasks differ, this contrast introduces a degree of conceptual inconsistency. The manuscript could be strengthened by proposing or discussing the potential development of a unified, generalizable framework that addresses multiple imaging tasks with minimal architectural or training modifications. Such an approach would enhance both the methodological contribution and the practical significance of the study.

2. The current description of the supervised training strategy, particularly for the multi-frame approach, could be expanded. It would be helpful if the authors provided more details on how the supervised model is trained, how multiple frames are incorporated, and what architectural or input–output adjustments are made to utilize temporal information effectively. In addition, the rationale for not adopting a multi-frame strategy for denoising, especially in the case of living-cell data, should be explained more clearly. Since the manuscript applies a model trained on fixed vimentin data to live-cell samples, providing a justification for this choice would be valuable. A multi-frame denoising approach for live-cell imaging could potentially offer more robust performance than a model trained exclusively on static data.

3. Including a quantitative comparison between the Fast-MoNaLISA multi-frame and single-frame modes would help demonstrate the specific benefits of the multi-frame approach. It would also be useful to clarify the method used for under-sampling across different time frames.

4. To improve reproducibility, the manuscript should provide more comprehensive training details, such as total training duration, network hyperparameters, machine specifications, and the runtime for testing. Including a training loss curve could also help illustrate network convergence. Furthermore, please specify the size and composition of the training dataset in greater detail. It would be reassuring to show evidence that the predicted outputs do not exhibit hallucination artifacts, especially for the unsupervised HDN model.

5. In figures, the ground truth data appears visually noisy. The authors are encouraged to clarify how quantitative evaluations remain meaningful when the ground truth itself contains significant noise. Discussing any preprocessing or validation methods that ensure the reliability of these evaluations would strengthen the interpretation of results.

6. In Figv1d, the authors claim that resolution of groundtruth is ~60 nm. However, it’s hard to believe this value from the figure. I recommend showing clear line profiles.

(Remarks on code availability)
Authors used codes that are already available in Github for example CARE, UNET-RCAN and HDN.

Reviewer #2

(Remarks to the Author)
This manuscript presents a valuable integration of deep learning techniques—specifically hierarchical denoising (HDN) and upsampling networks—into the MoNaLISA RESOLFT platform. The work convincingly demonstrates extended imaging duration (up to 5× longer) and increased acquisition speed (up to 4× faster) while preserving nanoscale resolution with experimental validation on cytoskeletal and mitochondrial dynamics. While the individual components (MoNaLISA, HDN, UNet-RCAN) are from previous publications, the systematic comparison of denoising methods and the innovative combination of subsampling with temporal-context networks constitute a meaningful advance for live-cell nanoscopy. My major points are as follows

1. The evaluation of all the networks are performed on a dataset of fixed vimentin. To strengthen the conclusion that HDN performs best, datasets containing more diverse structures (points, vesicles, et.) are required. Also, the authors note that CARE and UNet-RCAN perform similarly here, contrary to prior reports. More evidence would strengthen this finding.

2. The authors provide the code for model training and inference, which is valuable for replication. However, the HDN model, although published elsewhere, is not introduced in the main text. The authors may briefly introduce the model and comments on the reason of good performance. The dataset prepared for unsupervised training is not quite clear to me. I suggest the authors contains a dataflow graph on the supplementary notes.

3. How is the generalization of the HDN model? Denoising is a fundamental task for all fluorescent imaging. The method will be more valuable if it performs better on other types of datasets. It would also be helpful to discuss how transferable this model is across different microscopes, fluorophores, or cell types.

3. The use of 10–20% ON-switching is central to the photobleaching reduction. Could the authors comment on how this range was chosen? Was it empirically optimized based on rsEGFP2 switching kinetics or SNR thresholds? A discussion of whether even lower switching ratios (e.g., 5%) could be used and what the fundamental limits might be, would interest readers aiming to maximize imaging longevity.

4. Performance in context of other nanoscopy modalities: the achieved 5× longer imaging (e.g., 4 min at 5 s intervals) is impressive relative to standard RESOLFT, but how does it compare with other live-cell nanoscopy methods such as SIM, STED, or SOFI in terms of duration, speed, and photodamage? A short paragraph in the Discussion placing this work in the broader landscape would help readers appreciate its niche and advantages.

5. In the denoising section, UNet-RCAN showed some loss of resolution (wider FWHM). Yet, the same architecture is used for upsampling in Fast-MoNaLISA. Did the authors verify that the upsampling network does not degrade resolution compared to the Nyquist-sampled ground truth? A similar FWHM analysis for upsampled predictions (at least for S=50% and 25%) should be included to ensure that the gain in speed does not come at the cost of spatial fidelity.

6. Some grammar errors exist. Page 2: "…mitigate phototoxicity that arise…" should be "mitigates phototoxicity that arises…"; Figure 3 caption: "Quantification of the SNR levels are indicated…" should be "is indicated".

(Remarks on code availability)

Reviewer #3

(Remarks to the Author)
This manuscript presents a hybrid computational–experimental framework to extend the duration and increase the effective temporal resolution of live-cell RESOLFT nanoscopy, demonstrated using the MoNaLISA implementation. The authors introduce two complementary approaches: (i) prolonged imaging via low–ON-switching acquisition combined with deep-learning–based denoising (HDN/HDNsup), and (ii) accelerated acquisition ("Fast-MoNaLISA") by reducing scanning density and restoring subsampled images using a learned upsampling strategy, including a multi-frame variant that leverages temporal context. The technical work is carefully executed, the validation is thorough, and the biological demonstrations convincingly illustrate that these approaches enable observations that are difficult or inaccessible with standard MoNaLISA acquisition.
Overall, the study is strong and of clear interest. However, several issues related to framing, clarity, and interpretation currently limit its accessibility and broader impact for a general Nature Communications audience.
Major comments
Clarify the two main methodological contributions early and explicitly.
The Abstract and early Introduction do not clearly separate the two approaches and their respective claims. It would help to state explicitly that the manuscript presents (a) reduced ON-switching with computational restoration to extend imaging duration, and (b) reduced spatial sampling with restoration to increase acquisition speed, and to clearly associate

performance claims (e.g., longer time lapses versus faster imaging) with the corresponding method.
Strengthen articulation of the conceptual advance.
At first reading, the work can appear as an application or benchmarking of deep-learning approaches for microscopy. The manuscript becomes more compelling once it is clear that the authors propose (i) the use of HDN/HDNsup specifically because it preserves spatial resolution where other denoising methods show smoothing or loss of fine features, and (ii) a subsampling–restoration strategy tailored to MoNaLISA's coordinate-targeted acquisition, including randomized pixel selection to avoid structured artefacts. Highlighting these points earlier and more explicitly would help clarify the conceptual contribution beyond demonstrating performance gains.
Discuss robustness across labeling density and structural redundancy.
Most quantitative resolution analysis relies on vimentin filaments, which are a reasonable benchmark but also a favorable, highly redundant structure. Restoration performance is known to depend on labeling density and structural heterogeneity. Additional validation or a clearer discussion of how denoising and subsampled reconstruction behave for sparser or less redundant targets would strengthen the generality of the conclusions.
Clarify sources of reconstruction error at low SNR and aggressive subsampling.
The manuscript appropriately notes increased risk of "error predictions" and the need to avoid "patterned noise" at low sampling ratios. It would be useful to more clearly distinguish limitations arising from reduced sampling density itself, from restoration-model behavior (e.g., suppression of dim structures), and from any intrinsic constraints of MoNaLISA reconstruction under undersampling. This clarification is important for guiding interpretation of restored images and for assessing transferability to other modalities.
Summarize trade-offs between duration, temporal resolution, and restoration fidelity.
While the demonstrations are convincing, the manuscript would benefit from a more explicit summary of operating regimes. In particular, what ultimately limits prolonged imaging once ON-switching is reduced, and how sampling ratio Sshould be chosen in practice as a function of SNR and dynamics. The results suggest diminishing returns beyond S=50%and higher risk at S=25%unless temporal context is used; presenting this guidance more clearly would improve usability.
Tighten integration of biological demonstrations with methodological advances.
The biological examples are strong, but at times read primarily as proofs of capability. Explicitly linking each example to the specific technical advance that enables it would strengthen the narrative and clarify why these observations could not be obtained with conventional acquisition.
Minor comments
Terminology around "speed" should be used consistently (acquisition time per frame vs effective temporal resolution vs volume rate).
A brief limitations paragraph summarizing known failure modes (e.g., smoothing, increased error predictions at low SNR or low S, suppression of dim vesicles in single-frame predictions) would be valuable for prospective users.
Clearer separation between validation figures and biological application figures would aid readability.

(Remarks on code availability)

Version 1:

Reviewer comments:

Reviewer #1

(Remarks to the Author)
The authors clearly addressed all the concerns I raised. I expect this work would be helpful for fast long-term RESOLFT imaging as well as other imaging modalities.

(Remarks on code availability)

Reviewer #2

(Remarks to the Author)
The authors have addressed all my concerns in the revised manuscript. I recommend publication in its current form.

(Remarks on code availability)

Reviewer #3

(Remarks to the Author)
The two complementary deep learning strategies presented — HDN-based denoising for extended imaging duration and Fast-MoNaLISA subsampling restoration for increased frame rate — represent a meaningful and valid technical advance that meaningfully pushes the experimental boundaries of live-cell RESOLFT nanoscopy.

The authors have responded thoroughly to my comments, and the manuscript is dramatically improved from the original submission. The addition of HIV Gag-IM virus-like particle data, the clarified conceptual framework distinguishing the

denoising and upsampling tasks, the expanded training details, and the discussion of failure modes and operating regimes all substantially strengthen the work.

(Remarks on code availability)

Open Access This Peer Review File is licensed under a Creative Commons Attribution 4.0 International License, which permits use, sharing, adaptation, distribution and reproduction in any medium or format, as long as you give appropriate credit to the original author(s) and the source, provide a link to the Creative Commons license, and indicate if changes were made.
In cases where reviewers are anonymous, credit should be given to 'Anonymous Referee' and the source.
The images or other third party material in this Peer Review File are included in the article's Creative Commons license, unless indicated otherwise in a credit line to the material. If material is not included in the article's Creative Commons license and your intended use is not permitted by statutory regulation or exceeds the permitted use, you will need to obtain permission directly from the copyright holder.
To view a copy of this license, visit https://creativecommons.org/licenses/by/4.0/

Point-by-point responses to reviewers of the manuscript :

RESOLFT time lapse imaging empowered by deep learning

Minet et al

We thank the reviewers for their constructive comments. In response, we performed new experiments and revised the manuscript to improve its clarity and consistency.

First, we added new denoising and up-sampling data on HIV Gag-IM virus-like particles (Fig. 1e-f, Fig. 3f-g) thus validating both of our methods on a sparse, dot-like structure. We also extended the validation of the temporal training by comparing single-frame and multi-frame predictions on three different structures: mitochondria (Fig. 4c–e), vimentin filaments (Fig. 4f–h), and Gag-IM particles (Supplementary Fig. S14).

We also expanded the description of HDN and clarified how it enables both supervised and unsupervised denoising (Main Text, Supplementary Note 2) and clarified the overall manuscript by better explaining the use of different network architectures for denoising and upsampling. Importantly, we clarified why these two tasks behave differently with respect to ground-truth's quality (Supplementary Note 1; Supplementary Figs. S1 and S11).

Finally, we added further training details, with discussion of practical operating regimes and failure cases. We believe these revisions substantially strengthen the manuscript and address the reviewers' concerns.

Below is a point-by-point response to the comments of each Reviewers, with the comments of the Reviewers in black and our responses in blue.

REVIEWER COMMENTS

Reviewer #1 (Remarks to the Author):

Minet et al. report deep learning based denoising methods for RESOLFT imaging, low-power nanoscopy suitable for live-cell imaging. Multiple approaches are demonstrated to achieve this goal, including supervised/unsupervised denoising, down/upsampling and multiframe training. The authors clearly show that their methods could benefit fast long-term RESOLFT imaging. Although not presented, it seems to be straightforward to apply this method to other live-cell super-resolution imaging techniques. Below are minor comments which would be greatly helpful to strengthen the manuscript.

1. The manuscript would benefit from improved structural clarity. The authors present two or three deep learning models addressing different tasks, denoising and up-sampling, but the use of distinct architectures for each somewhat reduces the methodological coherence and overall impact of the work. For example, the HDN model is chosen over the U-Net RCAN for denoising due to the smoothing artifacts observed in the latter. However, the same U-Net RCAN is employed in the up-sampling section without further discussion of these smoothing effects. Although the two tasks differ, this contrast introduces a degree of conceptual inconsistency. The manuscript could be strengthened by proposing or discussing the potential development of a unified, generalizable framework that addresses multiple imaging tasks with minimal

architectural or training modifications. Such an approach would enhance both the methodological contribution and the practical significance of the study.

We acknowledge that the first version of the manuscript did not clearly explain why each architecture was chosen, which was confusing. The reason behind these choices lies not in the architecture itself, but in the nature of the ground truth used for training. Indeed, for the denoising task, we found that supervised networks display a smoothing effect as the quality of the GT decreases. HDNsup allows this effect to be partly compensated by tuning the β_{KL} weight, but it still oversmooths when the GT quality degrades.

This has now been revised to clearly explain why UNet-RCAN fails in supervised denoising, and why it works for upsampling. This is first explained concisely in the main text, and then in more detail in Supplementary Note 1, with support from Supplementary Figs. S1 and S11.

We also agree that a common architecture for both tasks would be beneficial. However, an upsampling version of HDN does not currently exist, and we prioritized reconstruction quality over using a unified architecture, even if this required two different frameworks. Importantly, both methods are integrated within the same code structure, which minimizes the impact on users, who only need to choose the task: “denoising” or “upsampling”.

Of course, developing such a unified framework would be very useful in the future, especially if it could incorporate knowledge of the noise model into the upsampling task and thereby couple upsampling with denoising more effectively. However, this lies outside the scope of the present work. We have therefore proposed this direction as future work in the Discussion.

2. The current description of the supervised training strategy, particularly for the multi-frame approach, could be expanded. It would be helpful if the authors provided more details on how the supervised model is trained, how multiple frames are incorporated, and what architectural or input–output adjustments are made to utilize temporal information effectively. In addition, the rationale for not adopting a multi-frame strategy for denoising, especially in the case of living-cell data, should be explained more clearly. Since the manuscript applies a model trained on fixed vimentin data to live-cell samples, providing a justification for this choice would be valuable. A multi-frame denoising approach for live-cell imaging could potentially offer more robust performance than a model trained exclusively on static data.

The reviewer raises three points here: the need to clarify the temporal, or multi-frame, training; the reason why it was used only for the upsampling task and not for HDN denoising; and the reason why we used a model trained on fixed vimentin data rather than live vimentin data.

For the first point, the difference between single-frame and multi-frame training is actually quite minimal. We keep the same architecture and provide the temporal frames as input channels. We found that this already substantially improved upsampling performance without requiring time-specific additions to the architecture, which is why the initial manuscript did not explain this in more detail. However, we agree that this needed to be clarified. The temporal training now has its own full figure (Main Fig. 4), showing the training setup and representative results. In addition, a paragraph has been added to the Methods section to clearly state how the multi-frame data are prepared and fed to the network. We hope that this clarifies this point for readers.

For the second point, it is true that temporal denoising could be very exciting and could potentially lead to even better results. However, there are two reasons why we did not pursue it in this work. First, for HDN, we deliberately chose to use a published and well-established approach, for which no version currently exists that can natively reason over time. While we agree that leveraging temporal information is a logical and promising idea, no such extension of HDN currently exists. Developing a fundamentally new temporal denoising framework would be valuable but would also be outside the scope of this publication. Second, for supervised methods, temporal version would require an experimental strategy to acquire paired noisy-clean timelapses in live cells, which is very challenging. One potential approach would be to perform two acquisition cycles per pixel position: one with low ON-switching and one with high ON-switching, to minimize motion between the input and GT, pixels-to-pixels. However, this would require an entirely new acquisition scheme from a hardware perspective.

For the last point, the reason for using a network trained on fixed cells to denoise the live vimentin dataset is simply that the supervised version of HDN performed slightly better at lower SNR (Supplementary Fig. S2). Therefore, using the supervised network trained on fixed cells allowed us to extend the timelapse length by a few additional frames. A sentence has now been added to the main text to explain this more clearly.

3. Including a quantitative comparison between the Fast-MoNaLISA multi-frame and single-frame modes would help demonstrate the specific benefits of the multi-frame approach. It would also be useful to clarify the method used for under-sampling across different time frames.

We agree that the previous version did not clearly compare single-frame and multi-frame predictions. We now provide this comparison for three different structures: mitochondria, Gag virus-like particles, and vimentin filaments. For each structure, we present visual examples of the improvement provided by multi-frame prediction, such as improved resolution of an MDV, better separation between two Gag particles, or improved filament continuity. We also provide quantitative measurements, including PSNR and SSIM for several temporal windows, as well as FRC curves comparing single-frame and multi-frame predictions for the selected temporal window. These results are presented in Main Fig. 4 and Supplementary Fig. S14.

4. To improve reproducibility, the manuscript should provide more comprehensive training details, such as total training duration, network hyperparameters, machine specifications, and the runtime for testing. Including a training loss curve could also help illustrate network convergence. Furthermore, please specify the size and composition of the training dataset in greater detail. It would be reassuring to show evidence that the predicted outputs do not exhibit hallucination artifacts, especially for the unsupervised HDN model.

frames.

We now provide full details of the training parameters, training times, and dataset composition in Supplementary Tables 1 to 3. The machine specifications have also been added to the Methods section. Example training curves for each main task, including denoising, single-frame upsampling, and multi-frame upsampling, have now been added in Supplementary Fig. S18.

Regarding hallucination artefacts in HDN, we provide examples of failure cases for vimentin in Supplementary Fig. S2, which shows the differences between the supervised and unsupervised

versions at lower SNR, and for Gag particles in Supplementary Fig. S5, where the ON-switching level is too low (~5%). What we observed is that failure cases tend to correspond to missing information, such as a missing filament or a faint particle, rather than hallucination of non-existing structures.

5. In figures, the ground truth data appears visually noisy. The authors are encouraged to clarify how quantitative evaluations remain meaningful when the ground truth It self contains significant noise. Discussing any preprocessing or validation methods that ensure the reliability of these evaluations would strengthen the interpretation of results.

Experimental ground truths are never perfect and will always exhibit residual levels of noise, and it is indeed important to validate that pixel-wise metrics such as PSNR and SSIM remain meaningful in this context. To this end, we performed a small experiment, reported in Supplementary Fig. S17, where we show two things. First, although the absolute PSNR and SSIM values depend on the quality of the GT, relative comparisons between test images remain meaningful when the same reference is used, as noisier images consistently yield lower metric values independently of the GT quality. Second, PSNR and SSIM values calculated with a noisy GT are always lower than those calculated with a clean GT. This means that they represent a lower bound of the prediction quality.

6. In Figv1d, the authors claim that resolution of groundtruth is ~60 nm. However, it's hard to believe this value from the figure. I recommend showing clear line profiles.

We agree that the images alone are not enough to claim this resolution. Multiple line profile examples are now shown both in supplementary figure S1 and S12. We also hope that the additional virus-like particles images in the main Figure 1, displaying both confocal diffraction limited images and RESOLFT images help appreciate the resolution.

Reviewer #1 (Remarks on code availability):

Authors used codes that are already available in Github for example CARE, UNET-RCAN and HDN.

Reviewer #2 (Remarks to the Author):

This manuscript presents a valuable integration of deep learning techniques—specifically hierarchical denoising (HDN) and upsampling networks—into the MoNaLISA RESOLFT platform. The work convincingly demonstrates extended imaging duration (up to 5× longer) and increased acquisition speed (up to 4× faster) while preserving nanoscale resolution with experimental validation on cytoskeletal and mitochondrial dynamics. While the individual components (MoNaLISA, HDN, UNet-RCAN) are from previous publications, the systematic comparison of denoising methods and the innovative combination of subsampling with temporal-context networks constitute a meaningful advance for live-cell nanoscopy. My major points are as follows

1. The evaluation of all the networks are performed on a dataset of fixed vimentin. To strengthen

the conclusion that HDN performs best, datasets containing more diverse structures (points, vesicles, et.) are required. Also, the authors note that CARE and UNet-RCAN perform similarly here, contrary to prior reports. More evidence would strengthen this finding.

We agree that validation on a single structure is not sufficient to claim that one network performs best in all cases. The initial focus on vimentin filaments came from the possibility of directly assessing spatial resolution by measuring the size of single filaments, which are smaller than the RESOLFT resolution. Such measurements are not as straightforward on other structures.

To strengthen the validation, we have now added experiments on a much sparser structure: HIV Gag-IM virus-like particles. This dataset allows us to test HDN on dot-like, less redundant structures. We show that HDN accurately restores these particles while preserving the super-resolved details observed in the high-SNR RESOLFT images, which is not the case for other unsupervised methods (Main Fig.1). We also added additional validation of the upsampling approach on mitochondria and Gag particles. Together, these additions provide broader evidence that the proposed methods of this study are not limited to redundant filamentous structures.

Concerning UNet-RCAN, our intention was not to suggest that CARE, based on a U-Net architecture, and UNet-RCAN are equivalent in performances. In fact, in the second part of the manuscript, we found that UNet-RCAN performs better than a standard U-Net for the upsampling task. However, for supervised denoising, we found that performance is largely limited by the quality of the GT. Under these conditions, both CARE and UNet-RCAN are affected by smoothing when the GT quality decreases, whereas HDNsup mitigates this effect slightly more effectively, thanks to the possibility of fine-tuning the multi-solution latent space diversity. Thus, our finding that CARE and UNet-RCAN are performing similarly in this context is very specific to our datasets. We have now modified the main text to explain this more clearly, with a more detailed discussion in Supplementary Note 1 and supporting data in Supplementary Fig. S1.

2. The authors provide the code for model training and inference, which is valuable for replication. However, the HDN model, although published elsewhere, is not introduced in the main text. The authors may briefly introduce the model and comments on the reason of good performance. The dataset prepared for unsupervised training is not quite clear to me. I suggest the authors contains a dataflow graph on the supplementary notes.

We agree that the initial manuscript did not properly introduce HDN. This has now been corrected with additional paragraphs in the Main Text that clearly explain HDN and what sets it apart, as well as a more detailed explanation in Supplementary Note 2.

The data preparation for unsupervised HDN training does not involve a specific pairing strategy, unlike methods such as SN2N or N2V. Instead, unsupervised HDN training relies on the use of a probabilistic noise model. We believe that this point is now clarified by the revised explanation of how unsupervised HDN is trained.

3. How is the generalization of the HDN model? Denoising is a fundamental task for all fluorescent imaging. The method will be more valuable if it performs better on other types of

datasets. It would also be helpful to discuss how transferable this model is across different microscopes, fluorophores, or cell types.

HDN is generalizable to other microscopy techniques, and the original HDN paper demonstrated its performance on diverse structures, including Convallaria, fly wing, actin, and nuclei, and across multiple microscopy modalities, including confocal, two-photon, and spinning-disk microscopy. The only requirement is the acquisition of an experimental noise model, which can be done on any microscope by following the procedure described in the Methods section. We also mention the applicability of our method to other microscopy datasets in the Discussion of the main text.

In this study, however, we focus specifically on how HDN enables restoration of nanoscopy images such as RESOLFT images. We found that other existing methods, especially unsupervised methods, do not provide the same balance between denoising and preservation of spatial resolution. This is particularly important in our case, because our images are sampled near the Nyquist-Shannon limit (~30 nm scan steps for ~60 nm resolution). This sampling is necessary to image as fast as possible in living samples, but it also means that each pixel in the resulting image is crucial, with very little redundant information. As a consequence, any smoothing effect can directly deteriorate the image.

3. The use of 10–20% ON-switching is central to the photobleaching reduction. Could the authors comment on how this range was chosen? Was it empirically optimized based on rsEGFP2 switching kinetics or SNR thresholds? A discussion of whether even lower switching ratios (e.g., 5%) could be used and what the fundamental limits might be, would interest readers aiming to maximize imaging longevity.

The ON-switching levels were chosen empirically, by setting them as low as possible while maintaining an SNR level that allowed accurate predictions. While the optimal value depends on several factors, such as the biological structure, rsFP brightness, and labeling density, we found that 10–20% ON-switching works well with rsEGFP2 for all structures imaged in this study. We have added an example in Supplementary Fig. S5 showing prediction failure at ~5% ON-switching, and we now discuss this point in the main text.

4. Performance in context of other nanoscopy modalities: the achieved 5× longer imaging (e.g., 4 min at 5 s intervals) is impressive relative to standard RESOLFT, but how does it compare with other live-cell nanoscopy methods such as SIM, STED, or SOFI in terms of duration, speed, and photodamage? A short paragraph in the Discussion placing this work in the broader landscape would help readers appreciate its niche and advantages.

We thank the reviewer for this suggestion. One important feature to keep in mind when making this comparison is that RESOLFT can reach sub-100 nm resolution in live cells, whereas SIM and classical SOFI generally do not. STED, on the other hand, can of course reach similar resolution, and recent denoising approaches have made it gentler, similarly to what we propose here. However, its point-scanning implementation makes it difficult to reach ~1 s recording times over a full cell.

We believe that the strength of parallelized RESOLFT lies in its ability to perform whole-cell live imaging at ~1–2 Hz with sub-100 nm resolution. Our work further strengthens this capability by increasing imaging speed and duration while maintaining spatial resolution. We have now

added several paragraphs to the Discussion to explain this point and to place our work more generally in the context of other live cell nanoscopy techniques.

5. In the denoising section, UNet-RCAN showed some loss of resolution (wider FWHM). Yet, the same architecture is used for upsampling in Fast-MoNaLISA. Did the authors verify that the upsampling network does not degrade resolution compared to the Nyquist-sampled ground truth? A similar FWHM analysis for upsampled predictions (at least for S=50% and 25%) should be included to ensure that the gain in speed does not come at the cost of spatial fidelity.

This is an important point, and we thank the reviewer for raising it. We addressed this concern in two ways. First, we characterized the resolution of the upsampling predictions using both FWHM analysis of line profiles and FRC curves. We show in Supplementary Fig. S12 that the smoothing effect observed in the denoising task does not occur in the upsampling task, for any of the sampling ratios tested.

Second, we now provide an explanation for this difference in behavior between the two tasks. Although the same UNet-RCAN architecture is used, the training setup is fundamentally different. As mentioned in our response to the first comment, in the denoising task, the input is paired with an imperfect GT, which can result in a smoothing effect. In contrast, in the upsampling task, the input is generated directly from the GT. Therefore, even if the GT itself contains noise, that noise is intrinsically consistent with the input. This point is now briefly mentioned in the main text, fully explained in Supplementary Note 1, and demonstrated in Supplementary Fig. S11.

6. Some grammar errors exist. Page 2: "...mitigate phototoxicity that arise..." should be "mitigates phototoxicity that arises..."; Figure 3 caption: "Quantification of the SNR levels are indicated..." should be "is indicated".

We thank the reviewer for pointing this out. These errors have now been corrected.

Reviewer #3 (Remarks to the Author):

This manuscript presents a hybrid computational–experimental framework to extend the duration and increase the effective temporal resolution of live-cell RESOLFT nanoscopy, demonstrated using the MoNaLISA implementation. The authors introduce two complementary approaches: (i) prolonged imaging via low–ON-switching acquisition combined with deep-learning–based denoising (HDN/HDNsup), and (ii) accelerated acquisition ("Fast-MoNaLISA") by reducing scanning density and restoring subsampled images using a learned upsampling strategy, including a multi-frame variant that leverages temporal context. The technical work is carefully executed, the validation is thorough, and the biological demonstrations convincingly illustrate that these approaches enable observations that are difficult or inaccessible with standard MoNaLISA acquisition.
Overall, the study is strong and of clear interest. However, several issues related to framing,

clarity, and interpretation currently limit its accessibility and broader impact for a general Nature Communications audience.

Major comments

Clarify the two main methodological contributions early and explicitly.
The Abstract and early Introduction do not clearly separate the two approaches and their respective claims. It would help to state explicitly that the manuscript presents (a) reduced ON-switching with computational restoration to extend imaging duration, and (b) reduced spatial sampling with restoration to increase acquisition speed, and to clearly associate performance claims (e.g., longer time lapses versus faster imaging) with the corresponding method.

We thank the reviewer for this advice. We have reworked the Abstract and Introduction, as well as the general structure of the text, to better separate the two tasks.

Strengthen articulation of the conceptual advance.
At first reading, the work can appear as an application or benchmarking of deep-learning approaches for microscopy. The manuscript becomes more compelling once it is clear that the authors propose (i) the use of HDN/HDNsup specifically because it preserves spatial resolution where other denoising methods show smoothing or loss of fine features, and (ii) a subsampling–restoration strategy tailored to MoNaLISA's coordinate-targeted acquisition, including randomized pixel selection to avoid structured artefacts. Highlighting these points earlier and more explicitly would help clarify the conceptual contribution beyond demonstrating performance gains.

We agree that the initial manuscript did not clearly state our claims from the beginning. This has now been modified in the Abstract and Introduction.

Discuss robustness across labeling density and structural redundancy.
Most quantitative resolution analysis relies on vimentin filaments, which are a reasonable benchmark but also a favorable, highly redundant structure. Restoration performance is known to depend on labeling density and structural heterogeneity. Additional validation or a clearer discussion of how denoising and subsampled reconstruction behave for sparser or less redundant targets would strengthen the generality of the conclusions.

The focus on vimentin filaments came from the need to verify that the methods could preserve RESOLFT spatial resolution. Vimentin filaments are a useful structure for this purpose because single filaments are smaller than our spatial resolution, allowing line-profile analysis to be used as a direct way to assess resolution.

However, we agree that, in the context of deep learning, where restoration performance strongly depends on the structure, additional validation on different structures is important. We have now validated both denoising and upsampling on a new structure: HIV Gag-IM virus-like particles, which are much sparser and non-continuous. Upsampling validation is also clearly performed on mitochondria for the multi-frame temporal approach.

Clarify sources of reconstruction error at low SNR and aggressive subsampling.

The manuscript appropriately notes increased risk of "error predictions" and the need to avoid "patterned noise" at low sampling ratios. It would be useful to more clearly distinguish limitations arising from reduced sampling density itself, from restoration-model behavior (e.g., suppression of dim structures), and from any intrinsic constraints of MoNaLISA reconstruction under undersampling. This clarification is important for guiding interpretation of restored images and for assessing transferability to other modalities.

Concerning patterned noise, this is an artefact of the training setup that occurs if deterministic downsampling is used instead of randomized sampling. Thanks to the randomized downsampling strategy, this artefact does not occur during prediction. It was mentioned in the manuscript to justify the need for randomized subsampling. We have now changed the phrasing of this section to clarify this point for readers.

Concerning "intrinsic constraints of MoNaLISA reconstruction under undersampling", there is actually not really any specific artefacts coming from reconstruction of subsampled MoNaLISA data. This is because MoNaLISA reconstruction is very close to pure point-scanning reconstruction. Each pixel of the reconstructed image can be mapped to one specific scanning position, and this is why removing scan steps is equivalent to removing pixels in the final reconstructed image. This means that the method is transferable to any pure point-scanning method that maintains this simple reconstruction step, whereas methods such as image scanning microscopy would require adaptation. We have now added one sentence in the main text to emphasize this point when describing the Fast-MoNaLISA training setup, as well as another sentence in the Discussion about transferability.

Finally, concerning distinguishing between limitations coming from the subsampling itself and the model-behavior. In practice, these two sources of error are difficult to fully disentangle, because restoration-model errors arise precisely when the input contains too little reliable information, either because of low SNR or aggressive subsampling. However, we have now tried to separate causes when possible, and clarified this point in the manuscript. Reduced sampling density itself primarily limits the amount of spatial information available to the network: when S is too low, fine structures may simply not be sufficiently represented in the input image. This is not a model-specific behavior but a natural consequence of under-sampling. The restoration model can then introduce an additional bias by favoring the most likely reconstruction given the training data, which can lead to suppression of faint or ambiguous structures rather than hallucination of new ones. This is the behavior we observe, for example, for dim vesicles in single-frame Fast-MoNaLISA predictions, where temporal context helps recover structures that are otherwise treated as noise.

We have now added this clarification to the Discussion, emphasizing that errors at low SNR or low sampling ratios should be interpreted as the combined effect of limited input information and model regularization. We also explicitly state that, in our observations, the dominant failure mode is loss or suppression of weak structures, rather than creation of non-existing structures.

Summarize trade-offs between duration, temporal resolution, and restoration fidelity.
While the demonstrations are convincing, the manuscript would benefit from a more explicit summary of operating regimes. In particular, what ultimately limits prolonged imaging once ON-switching is reduced, and how sampling ratio should be chosen in practice as a function of SNR and dynamics. The results suggest diminishing returns beyond S=50%and higher risk at

S=25%unless temporal context is used; presenting this guidance more clearly would improve usability.

We have now added both intermediate conclusions for each section (denoising / upsampling single-frame / upsampling multi-frames), as well as a summary of the performances in the discussion.

Tighten integration of biological demonstrations with methodological advances.
The biological examples are strong, but at times read primarily as proofs of capability. Explicitly linking each example to the specific technical advance that enables it would strengthen the narrative and clarify why these observations could not be obtained with conventional acquisition.

We believe that the new structure of the figures and main text makes this link much clearer. The first part uses denoising to prolong MoNaLISA imaging, allowing us to monitor vimentin filaments, the actin meshwork, and mitochondrial dynamics for longer. The second part uses Fast-MoNaLISA to follow fast mitochondrial dynamics, both in 2D and volumetrically, which would be harder or impossible to observe with regular MoNaLISA acquisitions.

Minor comments
Terminology around “speed” should be used consistently (acquisition time per frame vs effective temporal resolution vs volume rate).

We thank the reviewer for this point. We now use the terminology consistently throughout the manuscript.

A brief limitations paragraph summarizing known failure modes (e.g., smoothing, increased error predictions at low SNR or low S, suppression of dim vesicles in single-frame predictions) would be valuable for prospective users.

This has now been added to the discussion parameter.

Clearer separation between validation figures and biological application figures would aid readability.

This has now been solved with a better separation between technical development and biological application. Figures 1,3 and 4 are used to explain and validate the methods, and Figure 2 and 5 is showcasing biological applications.
